# Forcing dataset updates impact regional but not global Earth radiation budget trends in the GFDL AM4 climate model

**DOI:** 10.1073/pnas.2611210123

**Published:** 2026-11-03

**Authors:** Chongxing Fan, David Paynter, Ryan J. Kramer, Vaishali Naik

**Affiliations:** ^a^ Program in Atmospheric and Oceanic Sciences, Princeton University, Princeton, NJ 08540; ^b^ Geophysical Fluid Dynamics Laboratory, National Oceanic and Atmospheric Administration, Princeton, NJ 08540

**Keywords:** Earth radiation budget, climate model, climate forcing, aerosol radiative effect

## Abstract

The availability of up-to-date forcing datasets is critical for reconciling Earth system model simulations with contemporary observations. With the newly released CMIP7 forcing dataset, which extends the historical record through 2021, this study provides a systematic evaluation of how using updated forcings over the “middle-of-the-road” SSP2-4.5 scenario forcings influences the simulated radiation budget in the GFDL AM4 climate model. The updated forcing dataset used in the atmosphere model does not improve the agreement between the simulated global-mean top-of-the-atmosphere radiation trends and the satellite-observed trends. Regionally, we identify pronounced shifts in radiative energy partitioning between atmosphere and surface. Specifically, during 2001–2021, accelerated reductions in sulfate and black carbon aerosols over East Asia drive a significant decline in atmospheric solar absorption that matches observations, while a substantial increase in organic aerosols at higher latitudes induces a localized increase in atmospheric absorption that does not match observations. The substantial influence of localized forcing changes on simulated regional climate underscores the necessity for routine updates of forcing datasets.

## Significance

To faithfully simulate and attribute climate change, global models rely on accurate, up-to-date dataset of human and natural climate drivers, collectively termed as forcings dataset. The previous standard dataset used to drive these models only extended through 2014. We evaluated whether using a newly updated forcing dataset, now extended through 2021, improves model simulation of Earth’s radiation budget. Globally, the update did not meaningfully improve the match between model and satellite trends. However, at the regional level, the updated data captured the effects of reduced air pollution over East Asia more accurately, while overestimating the effect of increased particles over Siberia. These findings highlight that routinely updating forcing datasets is essential for accurately representing regional climate changes.

Climate models are essential tools for understanding Earth system dynamics and generating reliable predictions and projections of future climate evolution (1). Historical simulations and comparisons with observational records enable researchers to assess model fidelity and attribute observed climate variations to specific forcing agents (2, 3). These simulations require forcing datasets as fundamental inputs. The forcing datasets, which include time-dependent external inputs like well-mixed greenhouse gases, ozone, aerosols, solar irradiance, and volcanic emissions, serve to drive climate model simulations. As scientific understanding of the spatial and temporal evolution of anthropogenic and natural processes advances, these datasets are extended and updated, in turn influencing model simulations of historical and future climate change. Here, we assess the impact of the updated forcing datasets developed in support of the Coupled Model Intercomparison Project Phase 7 (CMIP7) (4) on simulations of the Earth’s radiation budget over the 1979–2021 period.

Beginning with CMIP6, a common suite of forcing datasets have been developed in a coordinated manner by subject-matter experts external to modeling centers and delivered via input4MIPs on approximately 5 to 7-y cycles (4–6). These standardized datasets are used by the international climate modeling community to understand, attribute and project changes in the Earth System. A specific challenge is that updates to the full dataset suite do not occur annually, and therefore models rely on constructed forcing conditions from future, hypothetical emission scenarios to extend the historical datasets in the interim (7). This implies that the historical simulations do not necessarily reflect actual observed trajectories of these climate drivers. Beyond expanded temporal coverage, updated forcing datasets often incorporate bias corrections, higher spatial resolution, and improved feature representation. These updates can exert a profound influence on simulated climate states, as demonstrated in the analyses of the transition from CMIP5 to CMIP6 (8, 9).

In preparation for the ongoing CERESMIP (10) and CMIP7 (11) experiments, the input4MIPs team has recently published the updated forcing datasets through and beyond 2021 (4), which allows the simulations in both protocols to extend until the planned end year of 2021. It is crucial to investigate the difference between the CMIP7 forcing dataset and the combined CMIP6 historical and scenario forcing (referred to as CMIP6 for simplicity thereafter), as well as the impact of the forcing update on climate simulations. Aside from forcing uncertainty into the long future, another interesting question is whether it is sufficient to use a scenario-based forcing necessary for the models to simulate the real-world conditions, or whether it is necessary to provide up-to-date forcing regularly for climate models to run up to the near present.

This study aims to deliver an initial assessment of the impact of the CMIP7 forcing dataset, specifically greenhouse gas forcing, aerosol forcing, and natural forcing including volcanic aerosols and solar irradiance, on the Earth’s radiation budget based on GFDL AM4 atmosphere-only climate simulations. The Earth’s radiation budget is the fundamental driver of climate system dynamics. At the top of the atmosphere (TOA), the net balance of absorbed solar radiation (ASR) and outgoing longwave radiation (OLR), as well as its regional distribution, determines the global and local energy storage of the climate system. The surface radiation budget, as part of the surface energy budget, regulates surface temperature and governs heat transfer between atmosphere, land, and ocean, fundamentally driving local and regional weather phenomena. Therefore, analyzing the impact of forcing updates on Earth’s radiation budget serves as a primary indicator of the broader impacts on simulated climate states.

In addition, satellite observations provide a continuous record of the TOA energy budget through the present (12, 13), facilitating a direct comparison between model-simulated radiative fluxes and the observational records. In general, CMIP6 models underestimate the net TOA radiation trend over the last two decades when compared to the Clouds and the Earth’s Radiant Energy System (CERES) (14, 15) observations, regardless of whether they utilize fully coupled configurations or Atmospheric Model Intercomparison Project (AMIP) (16) simulations with prescribed sea surface temperatures and sea ice concentrations (17–21). This underestimation has been attributed to an insufficient TOA shortwave (SW) trend within the models. Beyond model physics uncertainty, there is little understanding of the extent to which forcing dataset uncertainty may affect these observed biases in the simulated radiation budget. In contrast, few studies have evaluated model-simulated surface radiative fluxes and atmospheric absorption (calculated as the difference between TOA and surface fluxes) against observational records.

In this study, we assess the impact of CMIP7 forcing updates on Earth’s radiation budget by first comparing the GFDL AM4 (22, 23) runs with CMIP7 forcing against control simulations that use the CMIP6 historical forcings extended to 2021 by the “middle-of-the-road” SSP2-4.5 future scenario forcings (*SI Appendix*, Figs. S1–S5) (24), which assumes the world does not shift markedly from historical patterns over the coming decades. Simulated TOA and surface radiative fluxes were compared to the CERES satellite observations. Despite the temporal coverage of the forcing dataset spanning from 1850 to near present, here our analyses focus on the CERES period of 2001–2021 and the full AMIP simulation period of 1979–2021. Here, we report that the CMIP7 forcing dataset minimally alters simulated global net TOA radiation trends and variability, which remain significantly below satellite observations (Section 1). However, it introduces a decrease in atmospheric SW absorption and a consequent increase in downwelling surface SW radiation, which is attributable to aerosols (Section 1). Regionally, a marked reduction in sulfate and black carbon over East Asia reduces atmospheric absorption and brings the model into closer alignment with observations (Section 2). While an abrupt increase in organic aerosols over higher latitudes—such as Siberia—occurs around 2020 and causes a corresponding increase in atmospheric SW absorption, satellite data does not fully support the magnitude of this shift (Section 2).

## 1. Impact of Forcing Update on Global Radiation Budget

Comparing the CMIP7 forcing dataset to CMIP6, the time series of GHG concentrations (including CO_2_, CH_4_, N_2_O, and various CFCs) closely align during the overlapping period (1979–2014). Post-2014, compared to the SSP2-4.5 projections, the updated forcing dataset reports slightly less CO_2_ and CH_4_, along with slightly more N_2_O and CFCs, by up to 3% in concentration relatively and consequently ~0.01 W/m^2^ in instantaneous radiative forcing (IRF; *SI Appendix*, Fig. S1). The input total solar irradiance in the CMIP7 forcing dataset increases by an average of 0.57 W/m^2^ relative to the CMIP6 dataset over the period of 1979–2021. The corresponding linear trend is adjusted from −0.16 W/m^2^/decade in the CMIP6 dataset to −0.11 W/m^2^/decade in the CMIP7 dataset (*SI Appendix*, Fig. S2). The updated stratospheric volcanic forcing dataset reports enhanced forcing for the historical eruptions including the 1991 eruption of Mount Pinatubo. The input SW AOD and LW AOD increase by up to 16% and 25% for this event, respectively. The updated forcing dataset also records small post-2014 eruptions (*SI Appendix*, Fig. S3). Significant revisions are found in the CMIP7 anthropogenic aerosol emissions dataset. The most notable features in the updated dataset include reversed black carbon emission trend from positive to negative over East Asia, increasing black carbon emission over higher latitudes, widespread increase of organic matter emission over high-latitude Northern Hemisphere due to biomass burning, and enhanced sulfate reduction since 2008 (driven by controls in East Asia) with a notable sharp dip around 2020 attributable to reduced ship emissions (*SI Appendix*, Figs. S4 and S5).

**Fig. 1.**
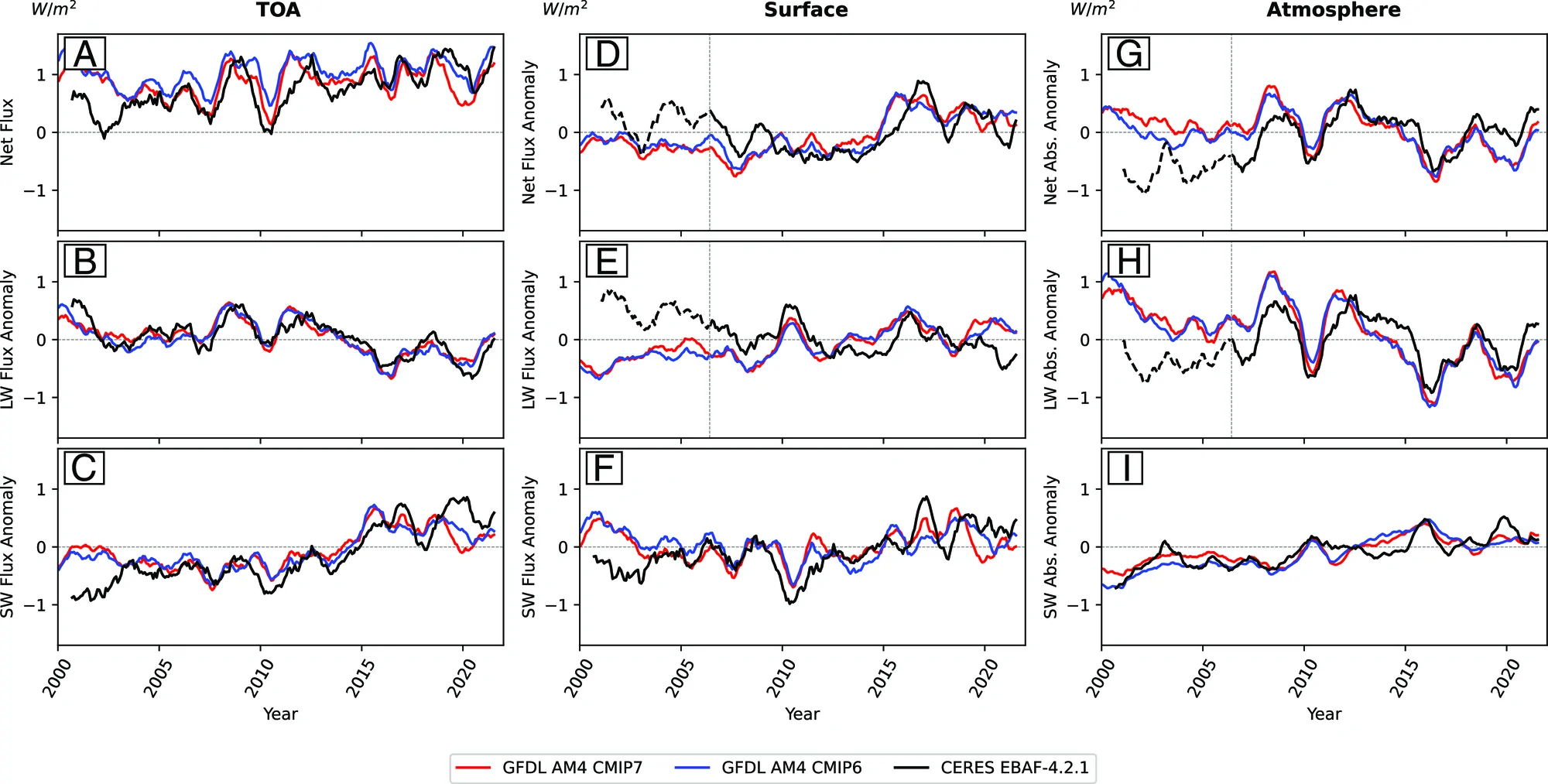
Monthly global mean anomalies of all sky TOA (*A*–*C*) and surface (*D*–*F*) radiative fluxes from CERES EBAF-4.2.1 observations (black) and ensemble mean of GFDL AM4 historical simulations forced by CMIP6 forcings (blue) and CMIP7 forcings (red). Atmospheric radiative absorption (*G*–*I*) is computed as the difference of net TOA flux and net surface flux. Net fluxes (*A*, *D*, *G*) are also decomposed into longwave components (*B*, *E*, *H*) and SW components (*C*, *F*, *I*). The long-term mean net TOA radiation is added back to the anomalies in panel (*A*) to show net energy imbalance. All variables are defined positive downward and smoothed by a 12-mo moving average for visualization purposes. The values in Fig. 1*B* are equivalent to minus outgoing longwave radiation (–OLR), and the values in Fig. 1*C* are ASR. Dashed lines indicate data with large uncertainty in the CERES EBAF surface product due to reanalysis issue before 2006.

Inspecting the period of 2001–2021 when CERES observations are available, after updating the forcing dataset to CMIP7, the ensemble of GFDL AM4 simulations reports a small decrease relative to the CMIP6 simulation in mean net TOA radiation imbalance (1.03 W/m^2^ to 0.86 W/m^2^; Fig. 1*A*), bringing it closer to the mean net TOA radiation imbalance in the CERES observations (0.76 W/m^2^). However, the ensemble mean TOA radiative flux trends are insignificantly modified by updating the forcing from CMIP6+SSP2-4.5 to CMIP7 (Fig. 1 *A*–*C*). The linear trend of net TOA radiation from 2001 to 2021 in the CMIP7 simulations is adjusted down from 0.18 ± 0.19 W/m^2^/decade in the CMIP6 simulations to 0.12 ± 0.22 W/m^2^/decade (Table 1). Regardless of the choice of the forcing datasets, the trend in modeled net TOA radiation remains considerably lower than the CERES-observed trends (0.44 ± 0.21 W/m^2^/decade; Fig. 1*A* and Table 1). As echoed in previous studies (17, 19), such an underestimation in net TOA radiation trend, which is widespread among many AMIP models, is primarily dominated by the underestimation of the TOA SW trend (Fig. 1*C*). CERES reports a strong increase in TOA solar radiation by 0.67 ± 0.22 W/m^2^/decade from 2001 to 2021, while the latest CMIP7 AM4 simulations produce only half of the CERES trend (0.35 ± 0.30 W/m^2^/decade; Table 1). Echoing Raghuraman et al. (17), such underestimation is more severe in the Southern Hemisphere (*SI Appendix*, Table S2), and compared to CMIP6 simulations, CMIP7 simulations produce less positive SW and net TOA flux trend in the Northern Hemisphere (*SI Appendix*, Table S2). It is important to note that even though the long-term trends do not compare well, the model captures the SW TOA variance in most of the period except the beginning (pre ~2003) and the end (post ~2017) periods (Fig. 1*C*). Both sets of simulations capture the LW TOA variance as well (Fig. 1*B*). The all-sky LW TOA radiation trend in the CMIP7 simulations becomes more negative than that in CMIP6 simulations and closer to the CERES-observed trend.

**Table 1.** Linear trends of TOA fluxes, surface fluxes, and atmospheric absorption in CERES observations and the ensemble mean of model simulations over the period of 2001–2021 in the unit of W/m^2^/decade

	CERES EBAF Ed4.2.1	GFDL AM4 CMIP7	GFDL AM4 CMIP6
TOA			
** **All–sky Net	0.44 ± 0.21	0.12 ± 0.22	0.18 ± 0.19
** **All–sky LW	−0.24 ± 0.22	−0.23 ± 0.22	−0.19 ± 0.35
** **All–sky SW	0.67 ± 0.22	0.35 ± 0.30	0.37 ± 0.25
** **Clear–sky Net	0.53 ± 0.15	0.12 ± 0.14	0.17 ± 0.12
** **Clear–sky LW	0.20 ± 0.16	−0.09 ± 0.19	−0.09 ± 0.23
** **Clear–sky SW	0.33 ± 0.13	0.22 ± 0.22	0.26 ± 0.20
Surface			
** **All–sky Net	0.02 ± 0.45*	0.40 ± 0.28	0.36 ± 0.28
** **All–sky LW	−0.37 ± 0.19*	0.30 ± 0.17	0.33 ± 0.16
** **All–sky SW	0.39 ± 0.22	0.10 ± 0.20	0.02 ± 0.19
** **Clear–sky Net	0.14 ± 0.34*	0.42 ± 0.25	0.38 ± 0.29
** **Clear–sky LW	0.13 ± 0.25*	0.48 ± 0.19	0.51 ± 0.18
** **Clear–sky SW	0.01 ± 0.19	−0.06 ± 0.15	−0.12 ± 0.22
Atmospheric Absorption			
** **All–sky Net	0.42 ± 0.33*	−0.28 ± 0.25	−0.17 ± 0.33
** **All–sky LW	0.13 ± 0.37*	−0.53 ± 0.35	−0.52 ± 0.39
** **All–sky SW	0.29 ± 0.12	0.25 ± 0.12	0.35 ± 0.20
** **Clear–sky Net	0.38 ± 0.33*	−0.30 ± 0.26	−0.21 ± 0.33
** **Clear–sky LW	0.07 ± 0.36*	−0.57 ± 0.37	−0.60 ± 0.39
** **Clear–sky SW	0.31 ± 0.14	0.27 ± 0.14	0.38 ± 0.22

The uncertainty values are 95% CI. Values with asterisks (*) denote items that are not trusted due to the MERRA-2 reanalysis issue in CERES EBAF surface flux products. See *SI Appendix*, Table S1 for the trend values over the period of 2006–2021.

The ensemble mean surface net radiative flux anomalies align closely between CMIP7 and CMIP6 simulations after the year 2000. Simulated surface LW fluxes in both cases track each other with the difference of linear trend less than 0.03 W/m^2^/decade (Fig. 1*E* and Table 1). The surface net SW radiation trend from 2001 to 2021 is adjusted up from 0.02 ± 0.19 W/m^2^/decade in the CMIP6 simulations to 0.10 ± 0.20 W/m^2^/decade in the CMIP7 simulations (Fig. 1*F*), which is mainly attributable to surface downward SW radiation trend increase from –0.19 ± 0.19 W/m^2^/decade to –0.08 ± 0.21 W/m^2^/decade. CMIP7 simulations show relatively higher anomalies of positive surface net SW flux around 2010 when compared to CMIP6 simulations. Both sets of simulations yield comparable values for surface net SW flux in more recent years. As a result, for the 2011–2021 period, CMIP7 simulations exhibit a notably weaker surface SW trend (0.08 ± 0.48 W/m^2^/decade) than CMIP6 simulations (0.38 ± 0.46 W/m^2^/decade).

Interestingly, CERES-derived surface radiative fluxes show a stark contrast to model simulations, with substantially different trends (Table 1). CERES reports a strong increase in net surface SW radiation (0.39 ± 0.22 W/m^2^/decade from 2001 to 2021; Fig. 1*F*) and a compensating strong increase in net outgoing surface LW fluxes (0.37 ± 0.19 W/m^2^/decade from 2001 to 2021; Fig. 1*E*), resulting in a near balance of net surface radiative flux. Our model simulations, however, predict increasing LW and SW radiation into the surface. It should be noted that CERES surface fluxes are not direct measurements; rather, they are derived from a combination of observations and radiative transfer calculations that relies on inputs from reanalysis (15, 25, 26). For instance, CERES-reported surface LW fluxes prior to 2006 are subject to uncertainties stemming from surface temperature discontinuities in the MERRA-2 reanalysis (27, 28). Consequently, these surface products may carry significantly higher uncertainty than their TOA counterparts. If discarding the first 5 y, CERES still reports an increase in outgoing surface LW fluxes of 0.22 ± 0.28 W/m^2^/decade, opposite in sign to model simulations (*SI Appendix*, Table S1).

For the full AMIP period of 1979–2021 (*SI Appendix*, Fig. S6), differences in simulated TOA fluxes between the CMIP6 and CMIP7 simulations are small with the notable exception of the 1991 Pinatubo volcanic eruption. For this particular event, CMIP7 simulations report an increased amount of reflected solar radiation of approximately 0.6 W/m^2^ (*SI Appendix*, Fig. S6*C*), which is partially offset by a 0.3 W/m^2^ reduction in OLR (*SI Appendix*, Fig. S6*B*). This is consistent with stronger stratospheric volcanic AOD in the updated forcing dataset (*SI Appendix*, Fig. S3). Linear trend differences remain statistically insignificant within the 95% CI. Regarding surface radiation budget, in addition to the volcanic eruptions, simulated surface net radiation is consistently lower in the CMIP7 simulations before 1997 by ~0.2 W/m^2^, attributable to surface SW radiation differences. For the full period of 1979–2021, GFDL AM4 CMIP7 simulations report a significantly stronger surface net radiation trend (0.38±0.17 W/m^2^/decade) than the CMIP6 counterparts (0.29±0.15 W/m^2^/decade) due to a more positive SW radiation trend (0.03 ± 0.15 W/m^2^/decade in CMIP7 compared to –0.06 ± 0.13 W/m^2^/decade in CMIP6; *SI Appendix*, Fig. S6*F*). Atmospheric radiative absorption, calculated by differentiating the net TOA radiative flux and the net surface radiative flux, shows a weaker increase in the CMIP7 simulations as a result (*SI Appendix*, Fig. S6*I*).

**Fig. 2.**
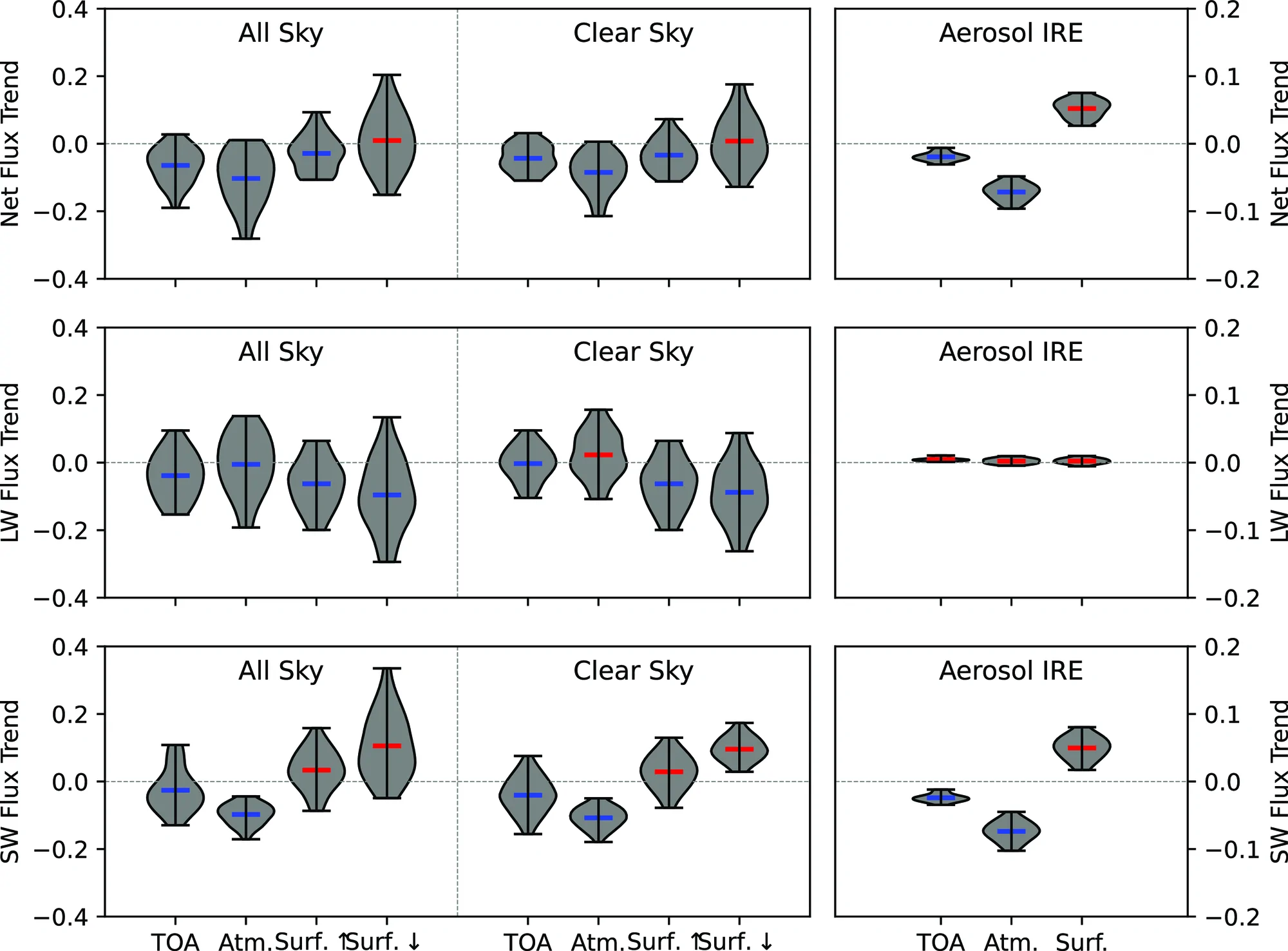
Differences in linear trends (2001–2021) of radiative fluxes from CMIP6 to CMIP7, including TOA radiative fluxes, atmospheric absorption (Atm.), and upward/downward surface radiative fluxes (Surf.). Each violin is computed by subtracting each of the five ensemble members of CMIP7 simulations by each of the five ensemble members of CMIP6 simulations (25 differences in total) and deriving the PDF of the differences. Positive values (red) mean that GFDL AM4 produces more positive trend when using CMIP7 forcing rather than CMIP6 forcing, and vice versa (blue). Longwave flux trend differences (middle row) and SW flux trend differences (bottom row) are calculated separately, and the net radiation trend is plotted in the upper row. Atm. is calculated by the difference of the net downward TOA flux and the net downward surface flux. Aerosol IRE is defined as the difference between all-sky flux and clean-sky flux (i.e., without aerosols).

To fully account for uncertainty from internal variability, trend differences between CMIP6 and CMIP7 were calculated using all possible combinations (25 pairs) of five ensemble members from each generation. Fig. 2 presents violin plots of the trend changes (CMIP7 − CMIP6) in all-sky and clear-sky radiative fluxes at both the TOA and surface, alongside changes in atmospheric absorption. Also included are trend differences for the aerosol instantaneous radiative effect (IRE). IRE is diagnosed online through a double call to the radiation module at each radiation time step, which computes fluxes with and without aerosols (i.e., clean sky) in the same simulation. As previously discussed, shifting from the use of CMIP6 to CMIP7 forcing in GFDL AM4 results in minimal changes to TOA flux trends. However, CMIP7 simulations exhibit a more negative trend in atmospheric SW absorption (change of –0.10 W/m^2^/decade for all-sky flux), alongside a pronounced increase in downward surface SW radiation (change of +0.11 W/m^2^/decade for all-sky flux) (bottom left panel of Fig. 2). Out of 25 pairs of simulations, 19 pairs indicate significantly less atmospheric SW absorption (at 95% confidence level) in the CMIP7 simulation than the corresponding CMIP6 simulation. Model ensemble spread in downward surface SW flux trend differences becomes narrower under clear-sky conditions, where the absence of cloud noise clarifies the signal. 21 out of 25 pairs show significantly negative trend differences in clear-sky atmospheric SW absorption. The reduction of atmospheric SW absorption and the resulting increase in downward surface SW radiation in CMIP7 simulations originate from the aerosol IRE contributions. Specifically, within CMIP7 simulations, the aerosol IRE contributes a trend increment of +0.05 W/m^2^/decade to surface SW radiation and –0.07 W/m^2^/decade to atmospheric SW absorption (bottom right panel of Fig. 2). At the TOA, the aerosol IRE also reflects significantly more SW radiation (0.02 W/m^2^/decade) in CMIP7. Differences in all-sky and clear-sky SW TOA fluxes remain generally negative; however, they lack statistical significance, likely due to noise introduced by the internal variability of water vapor and clouds across ensemble members.

## 2. Regional Radiative Flux Differences Attributable to the Updated Anthropogenic Aerosol Forcing

**Fig. 3.**
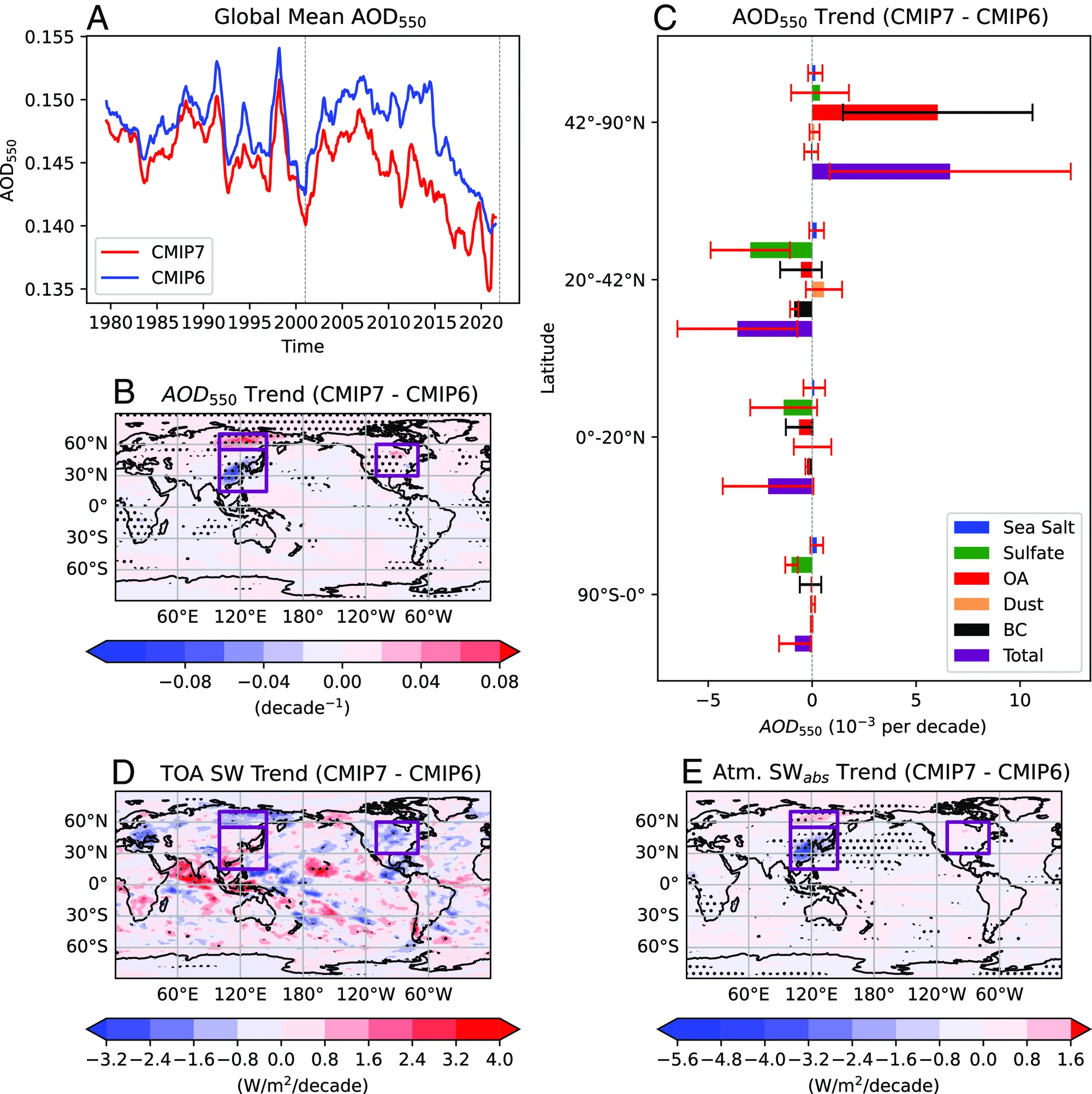
The impact of anthropogenic aerosol forcing updates on Earth’s radiation budget. (*A*) Time series of AOD at 550 nm (AOD_550_) computed by the GFDL AM4 model when using CMIP6 forcing (blue) and CMIP7 forcing (red). Both series have been smoothed by a 12-mo moving average for visualization purposes. (*B*) Spatial pattern of difference of AOD_550_ trend from 2001 to 2021 when CMIP7 forcing is used compared to CMIP6. Dotted region indicates significant trend differences at 95% confidence level. (*C*) Decomposition of AOD_550_ trend differences into different species of aerosols, including black carbon (BC), dust, organic aerosols (OA), sulfate, and sea salt, in the Southern Hemisphere and three same-area latitudinal bands in the Northern Hemisphere (i.e., 0°N–20°N, 20°N–42°N, and 42°N–90°N). Error bars indicate 95% CI. (*D*) Spatial pattern of the trend differences of net TOA SW radiation. (*E*) Spatial pattern of the trend differences of SW atmospheric absorption. The purple boxes indicate the three regions of interest that will be analyzed later in Section 2.

We now examine the relationship of the reduction of atmospheric SW absorption and the anthropogenic aerosol forcing updates from CMIP6 to CMIP7. The radiation module in GFDL AM4 calculates aerosol optical depth (AOD) and other optical properties based on input aerosol loadings of different species. These properties define the absorption and scattering characteristics of the atmosphere. Between 1979 and 2014, compared to the CMIP6 simulations, CMIP7 simulations report lower mean AOD by 0.002 in GFDL AM4 (Fig. 3*A*). Such lower AOD in CMIP7 relative to CMIP6 simulations is more prominent after the year 2008 (lower by 0.006). After 2014, forced by the SSP2-4.5 scenario projection, AOD in the CMIP6 simulations declines steadily with minimal interannual variability. In contrast, CMIP7 AOD exhibits significant fluctuations, including a sharp decrease in 2020 followed by a quick rebound after 2020 (Fig. 3*A*). This results from a combination of biomass burning in recent years and a remarkable decrease in sulfate emission from ships (*SI Appendix*, Fig. S4). Regionally, inspecting the recent 21 y (2001–2021), CMIP7 reports a much stronger decrease of AOD in East Asia (Fig. 3*B*) driven primarily by the reduced sulfate emission and secondarily by the reduced black carbon and organic aerosols (Fig. 3*C*). These forcing updates reflect substantial aerosol reductions resulting from emission policies adopted in East Asia (29). As a result, AOD trend in CMIP7 simulations aligns more closely with Moderate Resolution Imaging Spectroradiometer (MODIS)-observed trends in this region (*SI Appendix*, Fig. S7). On the other hand, compared to the CMIP6 forcing dataset, the CMIP7 dataset adjusts up the AOD trend over the Siberia region (Fig. 3*B*) with major contributions from increasing concentration of organic aerosols (Fig. 3*C*). This could be related to the wildfire emission in this region (*SI Appendix*, Fig. S5) (30).

The impact of anthropogenic aerosol forcing updates on all-sky TOA radiation is minimal even at the regional scale (Fig. 3*D*). However, the bipolar pattern of the AOD differences, characterized by reduced aerosols in East Asia and more aerosols over Siberia, translates into a corresponding pattern in atmospheric SW absorption, where East Asia sees less absorption and the East Siberia region sees more absorption (Fig. 3*E*). Because of the latitudinal distribution of the incoming SW radiation, the magnitude of the trend difference is larger over East Asia than over Siberia. When averaged globally, the decrease in atmospheric SW absorption is more dominant, explaining the decreasing trend in CMIP7 simulations (Fig. 2). To isolate the impact of individual forcing agents, we conducted single-forcing experiments in which greenhouse gases, aerosols, or natural factors (volcanic and solar) were varied while all other forcings remained fixed at 1850 levels. Only when historical variation of aerosol forcing is included in the simulations do the decrease of atmospheric SW absorption and the associated increase of downward surface SW radiation show up, suggesting that the trend differences in anthropogenic aerosol loading explain nearly all the trend differences in radiative fluxes between the two generations (*SI Appendix*, Figs. S8 and S9). While CMIP6 simulations show a global increase in atmospheric SW absorption, CMIP7 accurately captures the SW absorption decrease observed by CERES over East Asia during the last two decades (*SI Appendix*, Fig. S7). However, the atmospheric SW absorption produced by CMIP7 over East Siberia is considerably higher than that indicated by CERES observations (*SI Appendix*, Fig. S7).

**Fig. 4.**
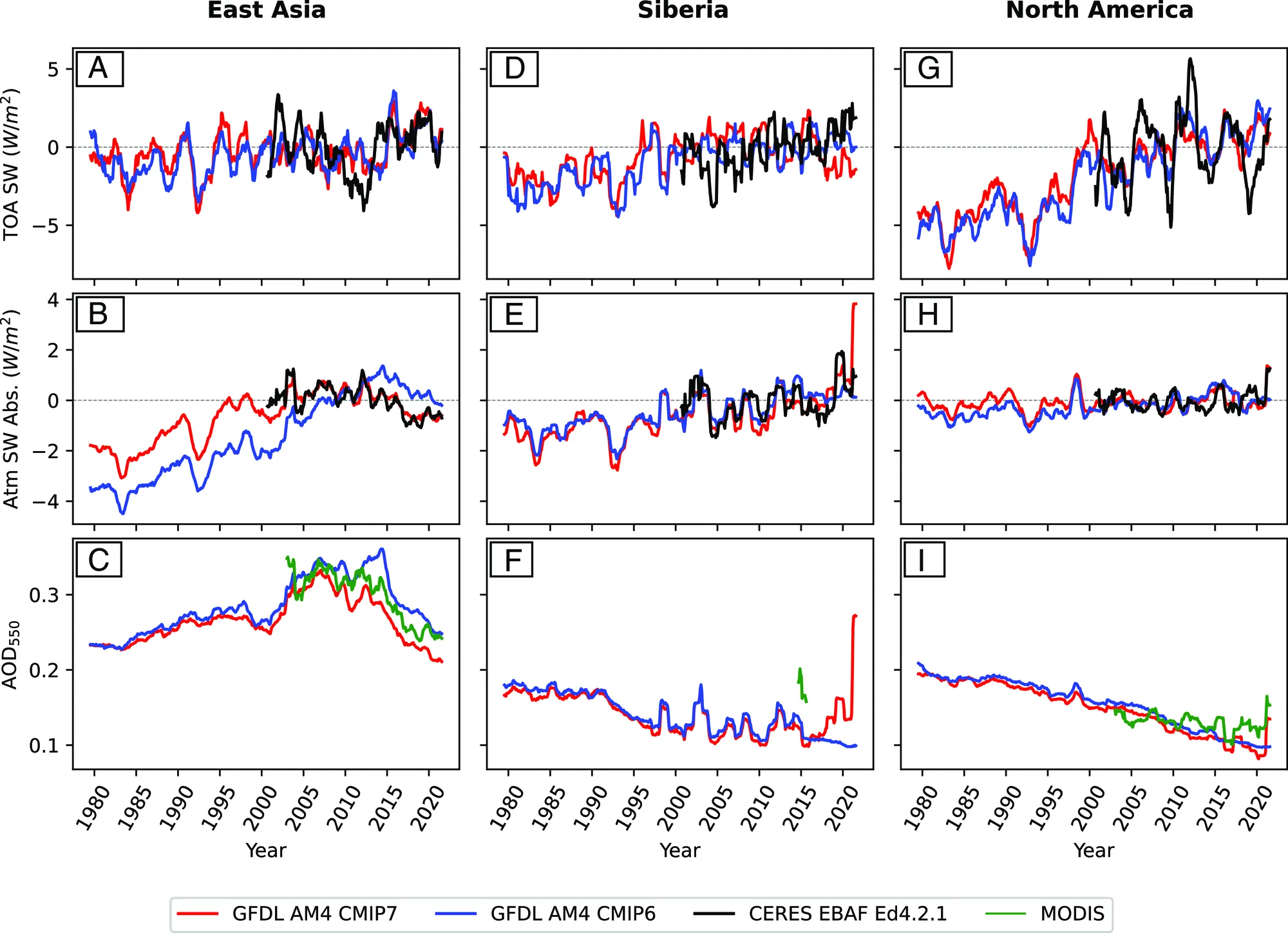
Monthly mean anomalies of net shortwave (SW) radiation at the top of the atmosphere (TOA), atmospheric SW absorption, and monthly mean aerosol optical depth (AOD) at 550 nm in GFDL AM4 simulations. Panels (*A*–*C*) show results for East Asia, (*D*–*F*) for East Siberia, and (*G*–*I*) for North America (regions identified in Fig. 3). Within each group, the panels show net SW radiation at the TOA, atmospheric SW absorption, and AOD at 550 nm, respectively. CERES-observed flux anomalies are shown as black lines in the first two rows of panels, and MODIS AOD is shown in the last row. A 12-mo moving average is applied to all time series for visualization purposes.

Now we focus on the three hot spots identified in Fig. 3: East Asia (15°N–55°N, 100°E–145ºE) where widespread AOD decrease and the corresponding atmospheric SW absorption decrease are observed in the CMIP7 simulations, East Siberia (55°N–70°N, 100°E–145°E) where large-scale AOD increase and atmospheric SW absorption increase are found, and a portion of North America (30°N–60°N, 110°W–70°W) that has witnessed AOD decrease over East Coast but also increasing wildfires in recent years. From the time series shown in Fig. 4*C*, both total AOD and absorbing AOD (*SI Appendix*, Fig. S10) over East Asia is lower by ~0.04 after approximately 2008 in the CMIP7 simulations, and such relatively low AOD continues after 2014. Aerosol reductions have distinct effects on radiative transfer: the decrease in absorbing aerosols directly lowers SW radiation absorption in the atmosphere, while the decrease in scattering aerosols increases the amount of SW radiation reaching the surface and shortening the path photons travel in the atmosphere (31). Overall, atmospheric SW absorption is observed to have decreased in the last 10 y (Fig. 4*B*). Because flux anomalies are calculated over the 2001–2021 period, decreasing SW absorption after 2008 is translated into larger flux absorption anomalies before 2008 in the time series (Fig. 4*B*). The linear trend of atmospheric SW absorption reverses from 0.86 ± 0.97 W/m^2^/decade to –0.38 ± 0.55 W/m^2^/decade. In this particular case, CMIP7 simulations report better agreement with MODIS-observed AOD (Fig. 4*C*) and CERES-derived SW absorption (Fig. 4*B*). However, both CMIP7 and CMIP6 simulations report mean TOA SW trend estimates that differ noticeably from the CERES value (0.96 ± 0.82 W/m^2^/decade in CMIP7, 0.68 ± 0.83 W/m^2^/decade in CMIP6, and 0.20 ± 1.15 W/m^2^/decade in CERES), despite the large uncertainties (Fig. 4*A*).

Over East Siberia, the more positive AOD trend in the CMIP7 simulations as seen from Fig. 3*B* is caused by AOD increase after 2015 (Fig. 4*F*). The SSP2-4.5 scenario forcing is unable to capture the large interannual variability of aerosol forcing. Meanwhile, CMIP7 forcing dataset reports a sharp increase in biomass burning emissions around 2020 (Fig. 3 and *SI Appendix*, Figs. S4, S5, and S11). The model forced by such an increase in aerosol loading produces an expected sharp increase in atmospheric SW absorption (Fig. 4*E*) and a remarkable decrease in net SW radiation at the TOA (Fig. 4*D*). Directly comparing model-simulated TOA SW radiation with CERES observations over high-latitude regions presents unique challenges due to large variability driven by complex cloud dynamics and surface albedo variations from snow and sea ice. Qualitatively speaking, CERES observations show a gentle upward trend in TOA SW radiation over East Siberia (1.34 ± 0.99 W/m^2^/decade), with no notable decreases like those seen in the simulations (Fig. 4*D*). The sharp increase of atmospheric SW absorption at the end of the time series is also absent from the CERES-derived values (Fig. 4*E*). Unfortunately, modeled AOD trends cannot be compared to MODIS observed ones due to lack of samples in high-latitude regions.

Over North America, CERES observations report a very large variability in TOA SW radiation with little linear trend, while the model simulations produce steady TOA SW increases in the last two decades with smaller interannual variation (Fig. 4*G*). Correspondingly, MODIS AOD has a flatter trend than the model-simulated AOD (Fig. 4*I*). Poor data sampling in MODIS observations may contribute to such discrepancies (*SI Appendix*, Fig. S7). Atmospheric SW absorption, however, agrees better between the CMIP7 simulations and the observations (Fig. 4*H*). Notably at the end of the time series, an abrupt increase in AOD was recorded by MODIS (Fig. 4*I*) and a corresponding increase in atmospheric SW absorption was reported by CERES (Fig. 4*H*). In this specific event, CMIP7 simulations reproduce the AOD increase in relative scale and accurately match atmospheric SW absorption anomalies in CERES observations.

## 3. Discussion

This study conducts a comprehensive TOA and surface radiation budget analysis and compares the results when using the up-to-date CMIP7 forcing datasets to the ones when using the CMIP6 forcing datasets extended by the middle-of-the-road scenario forcing dataset (i.e., SSP2-4.5) in GFDL AM4. Updating to CMIP7 forcing improves the model’s mean net TOA radiation imbalance, bringing it closer to CERES observations. However, it does not affect the simulated TOA radiation trend, and GFDL AM4 still significantly underestimates the observed trend in net TOA radiation with the bias stemming from the SW component. Within the Earth system, the model with CMIP7 forcing tends to produce less SW absorption in the atmosphere and more SW radiation into the surface by 0.11 W/m^2^ per decade on global average, especially in the last 15 y. Regionally, the differences of linear trends feature a strong dipole: a significant decrease of atmospheric SW absorption by up to 4 W/m^2^ per decade over East Asia and a moderate increase by 1 W/m^2^ per decade over East Siberia. These differences are attributable to the aerosol forcing updates. In the CMIP7 forcing dataset, reduced sulfate and black carbon emissions over East Asia allow more SW radiation reaching the surface and diminish the strength of atmospheric absorption, respectively. This effect outweighs the impact of increasing organic aerosol emission over Siberia, where the lower incident solar radiation at high latitudes limits their radiative impact.

Our findings suggest that TOA radiation budget in the AMIP simulations is minimally modified by the updates of the CMIP7 forcing dataset at both global and regional scales. At least in terms of the Earth’s radiation trends, the CMIP7 simulations align closely with the “middle-of-the-road” scenario in CMIP6 projections very well. Minor deviations in atmospheric concentrations in the real world—specifically a small decrease in CO_2_ and CH_4_, alongside a small increase in N_2_O and CFCs (*SI Appendix*, Fig. S1)—do not alter the simulated Earth’s radiation budget as represented by this specific scenario. The same applies to other forcing agents from the global perspective (*SI Appendix*, Figs. S2–S5). Our results suggest that for the global-mean radiation budget, extending the historical forcing with a middle-of-the-road scenario is largely sufficient, given that the SSP2-4.5 projections of well-mixed greenhouse gases, solar irradiance, and global-mean aerosol emissions remain close enough to the reported real-world evolution over the post-2014 interval. The global-mean adequacy of a scenario extension should not be assumed for longer extensions or for scenarios that diverge more strongly from realized emissions.

From the regional perspective, however, the updated forcing dataset incorporates the latest high-resolution real-world emission data, leading to substantial modifications in the regional flux trend. This is particularly evident for short-lived species characterized by high spatial and temporal heterogeneity. General underestimation of sulfate and black carbon reduction over East Asia and the missing sharp increase of organic aerosol over East Siberia and North America are prominent examples in the scenario forcing driven simulations. Consistent with physical intuition, these changes lead to corresponding radiation response in the AMIP models. The simulated atmospheric SW absorption agrees well with CERES-derived values in East Asia (Fig. 4*B*). The consequent less atmospheric SW absorption of up to 4 W/m^2^ per decade over East Asia is coincident with a slight increase in simulated precipitation and evaporation after 2008 (*SI Appendix*, Fig. S13). The reverse is true for the higher-latitude Siberia region, especially over the last 5 y. However, the sharp reduction of TOA and surface SW radiation over East Siberia around 2020 does not match the CERES records (Fig. 4). If the updated forcing data is accurate and reflects real-world conditions, these inconsistencies most likely reflect deficiencies in the model due to modeling challenges in the high-latitude region where variability in snow cover and cloud cover is significant. Future investigation on this particular issue is warranted. Regardless of the underlying reasons, it is crucial to regularly update forcing datasets to assess regional climate change in model simulations and, beyond the global Earth’s energy imbalance, to compare model-simulated radiation with observations at the regional scale and at the process level. This is powerful for identifying potential model issues, as regional signals can often be significantly stronger than their globally averaged counterparts.

Comparing AMIP simulations to the CERES observations, underestimation of net solar radiation at the TOA is expected to persist after updating the CMIP7 forcing dataset. The degree to which AMIP models in the upcoming CMIP7 experiment could yield results closer to the CERES-observed values is now contingent upon the advancements made in model physics since the previous generation of models. Meanwhile, although CMIP7 sticks to the use of PCMDI SST and sea ice dataset as in the CMIP6 experiment, new evidence shows that alternative SST and sea ice products exist that could produce stronger clear-sky TOA SW radiation in the AMIP simulations (*SI Appendix*, Fig. S12) (20). In addition to routinely extending the time series, future updates to input forcing datasets should also include evaluation of alternative forcing products and assess their uncertainty and impact to the model simulations.

This study investigates the radiative response of one particular AMIP model to the updated CMIP7 forcing. While the sign and spatial pattern of the response follow directly from the emission updates and should be robust, the quantitative values could vary depending on the model’s aerosol optical properties and its representation of aerosol–cloud interactions (*SI Appendix*, Figs. S14 and S15). The same experiments should be conducted across a range of other AMIP models to fully evaluate and understand how forcing updates affect AMIP simulations. One step further, unlike AMIP simulations where sea surface temperature and sea ice are prescribed by historical observations, these surface state variables will also respond to the forcing updates in the coupled model simulations, both in mean value and its spatial pattern. Feedback processes may be triggered to further adjust the radiation budget and induce associated climate response. Evaluations of the forcing updates in fully coupled Earth system models covering the full period since the preindustrial level (1850–2021) are thus valuable to provide a complete and comprehensive assessment of the CMIP7 forcing dataset and inspire future improvements to both model inputs and model physics.

## 4. Materials and Methods

### 4.1. CMIP Forcing Dataset.

CMIP forcing datasets provide a standardized set of inputs for participating models, ensuring that differences in results are attributed to internal model physics rather than different assumptions about external drivers. The recently released forcing dataset for use in CMIP7 DECK experiments consists of 12 components (4), five of which are used to force the AMIP runs in this study (Table 2).

**Table 2.** Details of CMIP6 and CMIP7 forcing datasets used in our simulations

	CMIP6	CMIP7
Anthropogenic short-lived climate forcer (SLCF)	CEDS-2017-05-18 (32)	CEDS-CMIP-2025-04-18 (33)
Open biomass burning emissions	VUA-CMIP-BB4CMIP6-1-2 (34)	DRES-CMIP-BB4CMIP7-2-1 (35)
Greenhouse gas concentrations (GHGs)	UoM-CMIP-1-2-0 (36)	CR-CMIP-1-0-0 (37)
Stratospheric volcanic SO_2_ aerosol optical properties	IACETH-SAGE3lambda-3-0-0 (38)	UOEXETER-CMIP-2-2-1 (39)
Solar irradiance	SOLARIS-HEPPA-CMIP-3-2 (40)	SOLARIS-HEPPA-CMIP-4-6 (41)
Sea surface temperature (SST) and sea ice (SI) boundary forcing	HadISST 2.4.0.0 SST and HadISST 2.2.4.0 SI (42)	
Ozone forcing	UReading-CCMI-1-0 (43)	

All other forcing remains the same between CMIP6 and CMIP7 simulations. CMIP7 continues to use the PCMDI-AMIP sea surface temperature and sea ice dataset as CMIP6; while the version has been updated and the records extended, these modifications are expected to yield negligible differences in model simulations. However, in this study, we used HadISST 2.4.0.0 SST and HadISST 2.2.4.0 SI (42) in all simulations instead of the recommended PCMDI-AMIP 1.1.10 dataset (44). In the particular case of GFDL AM4, the simulated clear-sky TOA SW trend is more consistent with the CERES observations when the HadISST 2.x forcing dataset is used (*SI Appendix*, Fig. S12) (20). Because the CMIP7 ozone concentrations had not yet been stably finalized at the time of simulation, we kept using the CMIP6 + SSP2-4.5 ozone forcing dataset in all simulations. GFDL AM4 uses static vegetation, so land use forcing dataset is not utilized here.

### 4.2. CERES EBAF Ed4.2.1.

The CERES Energy Balanced and Filled (EBAF) product offers observation-based monthly gridded radiative fluxes (14, 15). The source of data includes two CERES instruments onboard Terra since March 2000, with the addition of another two instruments onboard Aqua satellite after July 2002, and is switched to the sole CERES instrument onboard NOAA-20 satellite after April 2022. TOA radiative fluxes at the global scale are extrapolated and averaged with the aid of geostationary satellites (14). An objective adjustment of global-mean net fluxes is applied to the EBAF product to remain consistent with independent estimates of Earth’s energy imbalance derived from ocean heat content inventories (14). The calculation of surface radiative fluxes requires a combination of the direct TOA measurements and radiative transfer modeling with reanalysis inputs from the Goddard Earth Observing System (GEOS) (15).

We compared our model output TOA fluxes against CERES EBAF Edition 4.2.1 (CERES EBAF-4.2.1) from 2001 to 2021, consisting of 21 full years. The product provides two versions of clear-sky fluxes, one that averages only over clear-sky scenes, and the other that applies the cloud clearing algorithm to compute clear-sky fluxes for all regions. We opted for the latter as it aligns better with the definition of clear-sky fluxes in climate models. Regardless of the choice, the clear-sky TOA flux trends from 2001 to 2021 are similar (*SI Appendix*, Fig. S12).

### 4.3. MODIS AOD.

We used the Moderate Resolution Imaging Spectroradiometer (MODIS) Collection 6.1 Dark Target and Deep Blue products as the observational reference for AOD (45–47). Originally developed as a byproduct of atmospheric correction to improve land surface and ocean color measurements, the Dark Target algorithm has since been refined into a primary tool for quantifying global aerosol properties (48, 49). The retrieval is built on the physical principle that aerosols appear to brighten naturally dark scenes. Consequently, two distinct algorithms are developed: one optimized for the dark surfaces of the global ocean across visible and longer wavelengths, and another specifically for vegetated or dark-soiled land surfaces. AOD at 550 nm is then computed from radiative transfer model based on the aerosol retrievals. The Deep Blue product is designed to fill data gaps left by the Dark Target algorithm over bright land surfaces. By utilizing violet wavelengths, where most land surfaces exhibit low reflectance, the algorithm ensures these regions appear dark in this specific spectral range to the sensor for aerosol retrieval. Validation against the AERONET ground-based measurement demonstrates that the MODIS aerosol product shows a generally good performance with strong correlation and low error (45, 46, 50, 51).

Here, we used the monthly one-degree MODIS Level 3 product from Terra and Aqua satellites (i.e., MOD08_M3 and MYD08_M3). The combined Dark Target and Deep Blue AOD at 0.55 micron for land and ocean (AOD_550_Dark_Target_Deep_Blue_Combined_Mean_Mean) from the two products are averaged and analyzed.

### 4.4. GFDL AM4/LM4.

In this study, we ran AMIP-compatible simulations using the GFDL AM4/LM4 model (22, 23), a state-of-the-art atmosphere/land model with a ~100 km horizontal grid and 33 vertical levels. When compared to the CERES observations, GFDL AM4 simulates slightly more reflected SW radiation and less emitted LW radiation at the TOA (~1 W/m^2^) (22). Evaluation against a suite of surface-based and satellite observations demonstrates that GFDL AM4 shows good agreement with observed total AOD (52). Historical simulations were performed using two different sets of forcings: one based on CMIP7 and the other combining CMIP6 with SSP2-4.5, as described above. Except for the forcing datasets, the setup of these simulations is identical. Both simulations span the period from 1979 to 2021 and consist of five ensemble members each. For simplicity, these two simulation sets will be referred to as the CMIP7 and CMIP6 simulations hereafter.

To attribute the flux changes to different forcing agents, we designed and executed a series of single-forcing experiments in accordance with the CERESMIP protocol (10). The simulations include all forcing runs (amip), historical SST and sea ice only runs (amip-sst), aerosol forcing only runs (amip-aer), greenhouse gas only runs (amip-ghg), and natural forcing only runs (amip-nat). Details and results from the single-forcing experiments are discussed in the supplementary materials (*SI Appendix*, Figs. S8 and S9).

### 4.5. Statistical Analysis.

The analysis focuses on the monthly mean time series of TOA and surface broadband radiative fluxes. Global mean fluxes are computed by averaging the gridded fluxes weighted by grid point area. The model outputs are first regridded from their native resolution to a 1° × 1.25° latitude-longitude grid and then averaged using cosine-of-latitude weighting. CERES fluxes are averaged using geodetic weighting, consistent with the procedure used by the CERES team, while assuming that the Earth is an oblate spheroid instead of a sphere. These weights are provided on the CERES website (https://ceres.larc.nasa.gov/data/general-product-info/#geodetic-zone-weights-information). For model simulations, the ensemble mean was calculated by averaging the five individual ensemble members. This is used in all figures except Fig. 2 and *SI Appendix*, Figs. S8 and S9.

To calculate flux anomalies for both model output fluxes and CERES-observed fluxes, the monthly climatology over the period from 2001 to 2021 (or from 2006 to 2021 in Fig. 1 and *SI Appendix*, Fig. S6) is computed and subtracted from each time series. We used ordinary least squares (OLS) to estimate the linear trend. The trend uncertainties are estimated by a procedure adopted in previous Earth radiation budget studies (53) that accounts for autocorrelation in the OLS residual. The standard way to calculate the uncertainty of the OLS regression is via a *t*-distribution Wald test. The 95% CI (i.e., 2.5 to 97.5%) is given by

[1] $$CI_{\mathrm{OLS}}\;=\;\pm t_{\alpha ,N\;-\;2}\sigma_{\mathrm{SE}},$$

where $\sigma_{\mathrm{SE}}$ is the SE of the OLS trend estimate. Essentially, we calculate the autocorrelation function (ACF) of the OLS residuals and assess if it is significant at any lag k compared to the CI given by

[2] $$CI_{k}\;=\;\pm t_{\alpha ,N\;-\;k}\sigma_{k},$$

where $t_{\alpha ,N-k}$ is the student-*t* statistic at significance level α with N-k degrees of freedom, N is the number of samples, and $\sigma_{k}$ is the SD of the ACF at lag k. Here, we applied a 10 to 90% CI to allow for a less stringent test of autocorrelation. $\sigma_{k}$ is formulated as (54):

[3] $$\sigma_{k}^{2}\;=\;\frac{1}{N}\left(1\;+\;2\sum_{i=1}^{k-1}{\mathrm{ACF}}_{i}^{2}\right).$$

If ACF at any lag i exceeds the CI given by Eq. **2**, we substitute the sample size N by the effective sample size $N_{eff}$ following Gelman et al. (55):

[4] $$N_{\mathrm{eff}}\;=\;\frac{N}{1\;+\;2\sum_{t=1}^{m}ACF_{t}},$$

where m is the first lag that satisfies both $ACF_{m+1}\;<\;0$ and $ACF_{m+1}\;+\;ACF_{m+2}\;<\;0$. This method was applied to both the global mean monthly anomalies and to individual grid points to generate a spatial trend map. We used the SciPy implementation of the linear regression (linregress) to conduct the analyses (56). The resulting trends of CERES TOA fluxes are consistent with the ones reported in previous studies (53).

To account for the uncertainty associated with internal variability when comparing the CMIP7 and CMIP6 simulations, we calculated the differences for all 25 possible pairings, specifically between each ensemble member of the CMIP7 simulations and each member of the CMIP6 simulations. We applied the same linear trend analysis to each pair, reporting uncertainties as 95% CI. The violin plot illustrates the distribution of trend differences across the 25 pairs, representing uncertainty derived from internal variability. In the main text, we also evaluate the number of trend differences that reach statistical significance, which incorporates the uncertainty from the linear regression.

## Supplementary Material

Appendix 01 (PDF)

## Data Availability

CMIP7 forcing datasets are summarized in the input4MIPs documentation website (https://input4mips-cvs.readthedocs.io/en/latest/) and can be downloaded following the individual links. CERES EBAF products can be downloaded at https://ceres.larc.nasa.gov/data/. MODIS data are distributed by the Level-1 and Atmosphere Archive and Distribution System Distributed Active Archive Center (https://ladsweb.modaps.eosdis.nasa.gov/) (57, 58). The source code of GFDL AM4 is available via Zenodo (59). Code to reproduce the analysis is deposited in Zenodo (60). All other data are included in the manuscript and/or *SI Appendix*.
