# Supplementary material for: Forcing dataset updates impact regional but not global Earth radiation budget trends in the GFDL AM4 climate model
Source: Proc Natl Acad Sci U S A. 2026 Nov 3;123(45):e2611210123. doi: 10.1073/pnas.2611210123 (PMC13752350; doi:10.1073/pnas.2611210123)
Supplement: Supplementary file 1 [file pnas.2611210123.sapp.pdf]

PNAS

Supporting Information for
Forcing Dataset Updates Impact Regional but Not Global Earth Radiation Budget Trends in the GFDL AM4 Climate Model

Chongxing Fan*, David Paynter, Ryan J. Kramer, Vaishali Naik

* Corresponding Author: Chongxing Fan
Email: cxfan@princeton.edu

This PDF file includes:

Supporting text
Figures S1 to S15
Tables S1 to S2

Supporting Information Text

Overview

In the supplementary materials, Figs. S1–S4 present a comparison between CMIP6 and CMIP7 forcing datasets as inputs to our model simulations. Fig. S5 displays the 2001~2021 global mean time series and linear trends of aerosol loading, focusing on the geographical distribution of three species of interest. Fig. S6 complements Fig. 1 and plots the time series of the simulated and observed radiative fluxes over the full 1979~2021 period. Fig. S7 compares CMIP6- and CMIP7- simulated aerosol optical depth (AOD) and atmospheric solar absorption to MODIS and CERES observations, respectively. Figs. S8 and S9 present the results of single-forcing experiments. Fig. S10 reports both total AOD and absorbing AOD in our simulations, suggesting that changes in both scattering aerosols and absorbing aerosols contribute to the trend differences. Fig. S11 illustrates the time series of organic aerosol emissions over the three regions of interest and pinpoints the source of abrupt increase in AOD over East Siberia and North America. Fig. S12 demonstrates the impact of SST and sea ice forcing uncertainty on Earth's radiation budget (1). Fig. S13 plots the time series of precipitation and evaporation in model simulations. Figs. S14 and S15 compare the cloud fraction and cloud microphysical properties between the CMIP6 and CMIP7 simulations.

Single-Forcing Experiments

To attribute the flux changes to different forcing agents, we designed and executed a series of single-forcing experiments in accordance with the CERESMIP protocol (2). We analyzed and compared a total of 50 simulations (5 cases × 2 forcing datasets × 5 ensemble members).

- All forcings (All): Historical forcings with observed SST and sea ice.
- SST and sea ice only (SST): Historical SST and sea ice; all forcings fixed at 1850 levels.
- Greenhouse gases only (GHGs): Historical SST, sea ice, and well-mixed greenhouse gases; all other forcings fixed at 1850 levels.
- Aerosols only (Aer.): Historical SST, sea ice, and aerosol forcings; all other forcings fixed at 1850 levels.
- Natural only (Nat.): Historical SST, sea ice, solar irradiance, and volcanic emission; all other forcings fixed at 1850 levels.

Fig. S8 shows the linear trend of TOA and surface radiative fluxes from 2001 to 2021 in each ensemble member of all-forcing and single-forcing simulations using CMIP7 forcing datasets, and Fig. S9 shows the trend differences between CMIP7 simulations and CMIP6 simulations. Starting from the SST and sea ice only simulations, the TOA energy balance over the last two decades shows a large increase in outgoing longwave radiation by ~0.5 W/m^2/decade resulting from surface and tropospheric warming, which is partially offset by an increase in TOA SW radiation by ~0.25 W/m^2/decade resulting from sea ice reduction. As a result, net TOA radiation becomes increasingly negative (i.e., losing energy). Natural forcing agents (i.e., volcano and solar irradiance) slightly reduce the TOA SW radiation with time. Simulations that incorporated the historical trend of greenhouse gas concentrations demonstrate a substantial reduction in the OLR trend. This leads to a considerably smaller increase in OLR over the last 21 years. The inclusion of the decreasing aerosols also contributes to a moderate increase in SW radiation at the TOA. The historical simulations indicate an overall increase in net TOA radiation. The sum of trend increments from individual forcing terms is not necessarily equal to the trend differences between the all-forcing simulations and the SST and sea ice only simulations due to nonlinear interactions among the atmospheric components. At the surface, all simulations produce positive net surface radiation trends from 2001 to 2021. Like the case at the

TOA, natural forcing agents slightly adjust down the amount of solar radiation reaching the surface with time. Greenhouse gases amplify the amount of longwave flux into the surface considerably, while aerosol reduction induces an increasing amount of solar radiation into the surface.

As previously observed in Fig. 2, after the forcing dataset was updated from CMIP6 to CMIP7, the trend in net TOA radiation shows a small and insignificant negative trend difference from greenhouse gases and aerosols. Note that the linear trend of radiative fluxes could change from CMIP6 to CMIP7 simulations due to the differences in base state at the year 1850. From the perspective of surface radiation budget, the only notably significant trend difference from CMIP6 to CMIP7 simulations lies in clear-sky SW flux trend. The positive trend difference in clear-sky SW flux trend, as echoed in Fig. 2, originates from the aerosol forcing update, as the similar amount of positive trend difference only shows up in the aerosol only simulations (Fig. S9). In the longwave, aerosol forcing update also induces a small decrease of LW flux reaching the surface. Because of the large internal variability and nonlinear interaction between atmospheric components, these trend differences do not show up in the all-forcing simulations.

Cloud Fields in the CMIP6 and CMIP7 Simulations

Because GFDL AM4 represents aerosol–cloud interactions, part of the simulated radiative response to the forcing update is mediated by clouds rather than by direct aerosol extinction. Figs. S14 and S15 document the cloud amount and cloud microphysical fields in the two sets of simulations so that the GFDL AM4 response can be compared against other models as CERESMIP or CMIP7 results become available. In the global mean, differences in total, high, middle, and low cloud amount between the CMIP6 and CMIP7 simulations are small. Regionally, over East Asia, where sulfate and black carbon emissions are substantially reduced in CMIP7, the simulations produce slightly lower cloud amount (Fig. S14, right column), a significant decrease in vertically summed cloud droplet number concentration (CDNC), scattered increases in cloud-top effective droplet radius (r_{eff}), and a significant decrease in cloud liquid water path (LWP) (Fig. S15, right column). This is expected from weaker aerosol activation under reduced aerosol loading. Other regions do not show significant cloud property changes when using the CMIP7 forcing dataset.

Figures

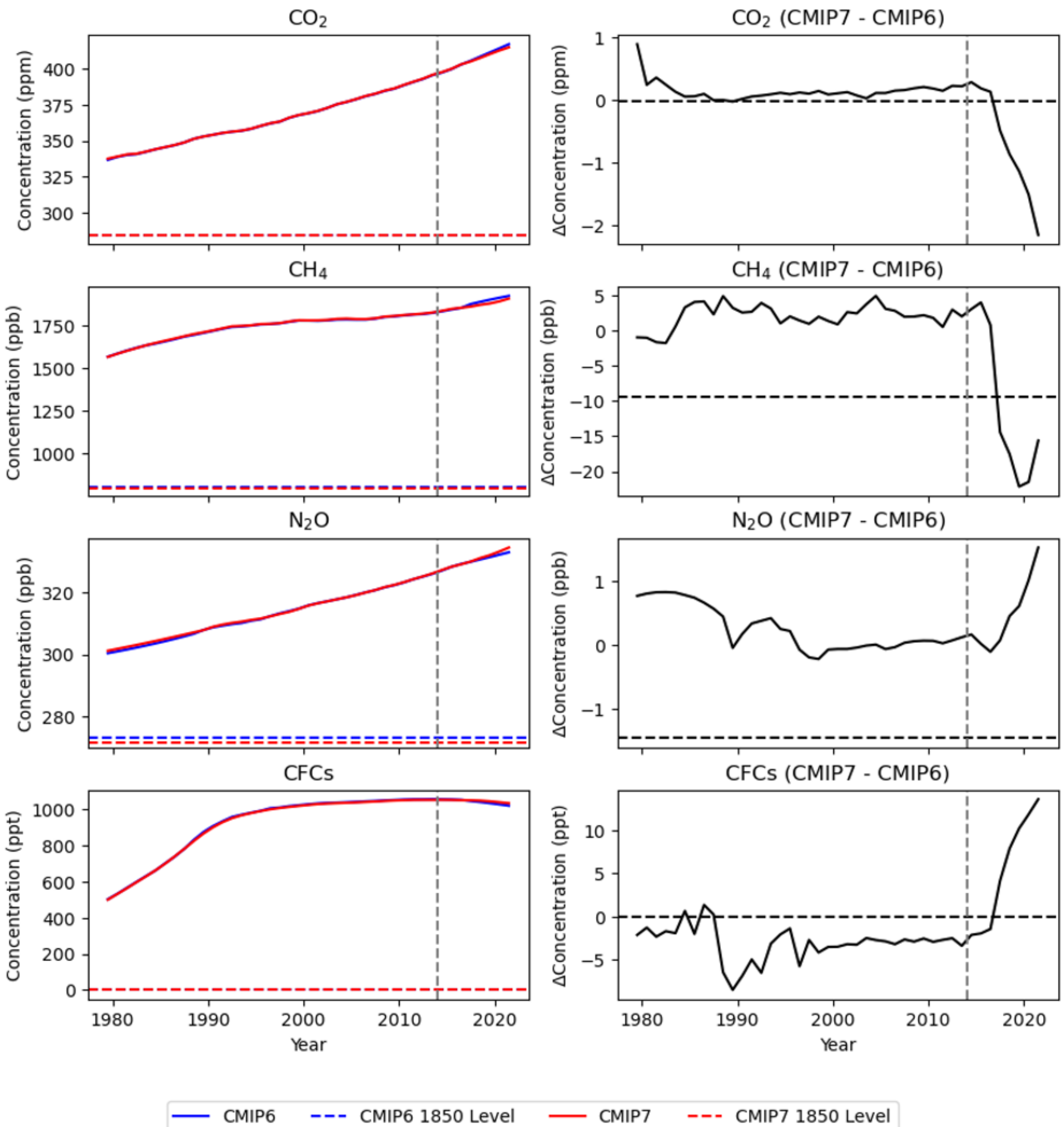

Fig. S1.
(left column) Global mean time series of greenhouse gas concentrations including carbon dioxide (CO_2), methane (CH_4), nitrous oxide (N_2O), and chlorofluorocarbons (CFCs) from 1979 to 2021 as inputs to the CMIP6 and CMIP7 simulations. The concentrations at the year 1850, which represent preindustrial conditions, are included as horizontal dashed lines. The gray vertical dashed lines mark the year 2014 after which CMIP6 simulations use SSP2-4.5 scenario forcing. (right column) the difference of greenhouse gas concentrations between the CMIP7 and CMIP6 forcing datasets.

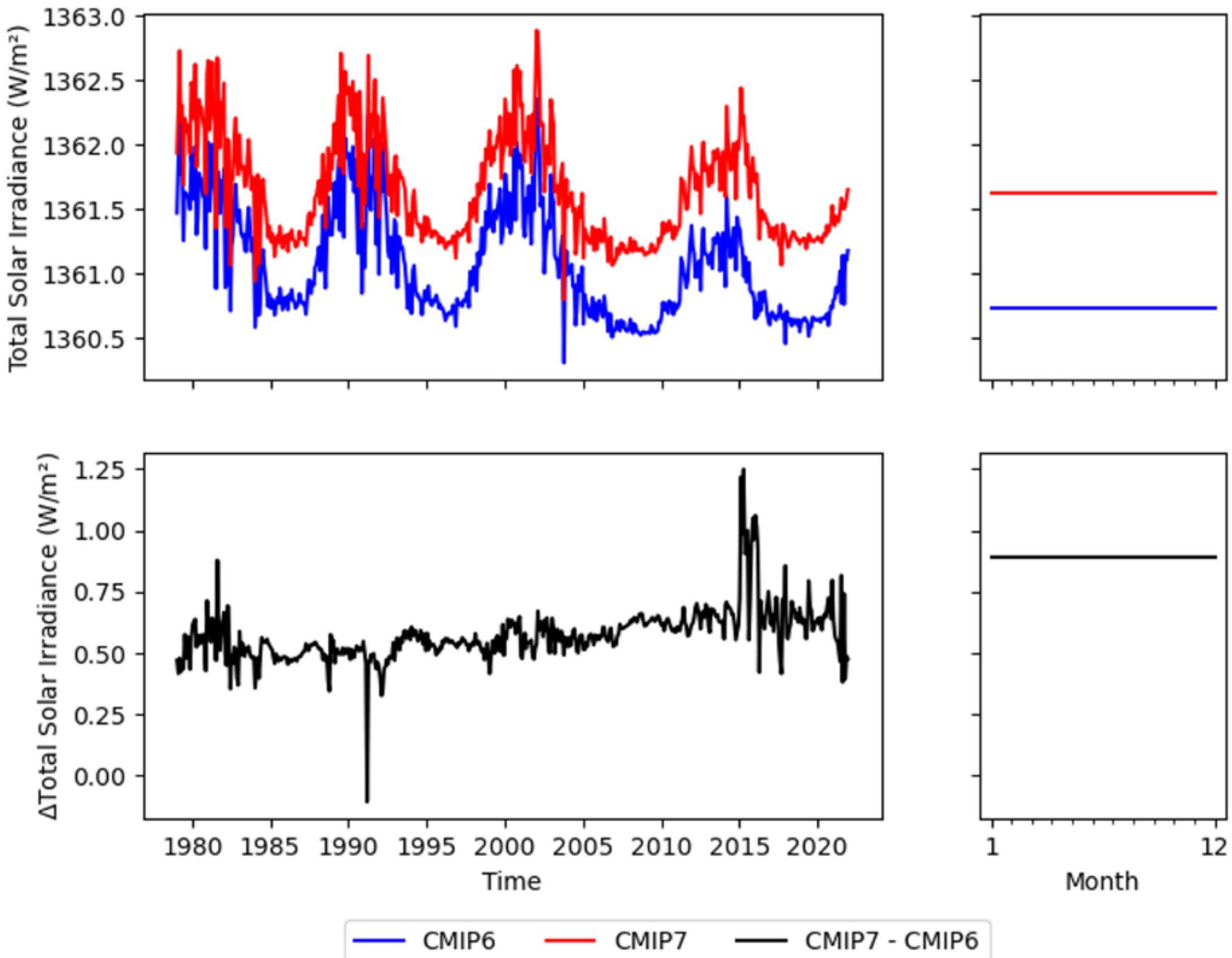

Fig. S2.
Global mean time series (left) and the 1850 climatology (right) of total solar irradiance as inputs to the CMIP7 and CMIP6 simulations (top), as well as the differences between the two.

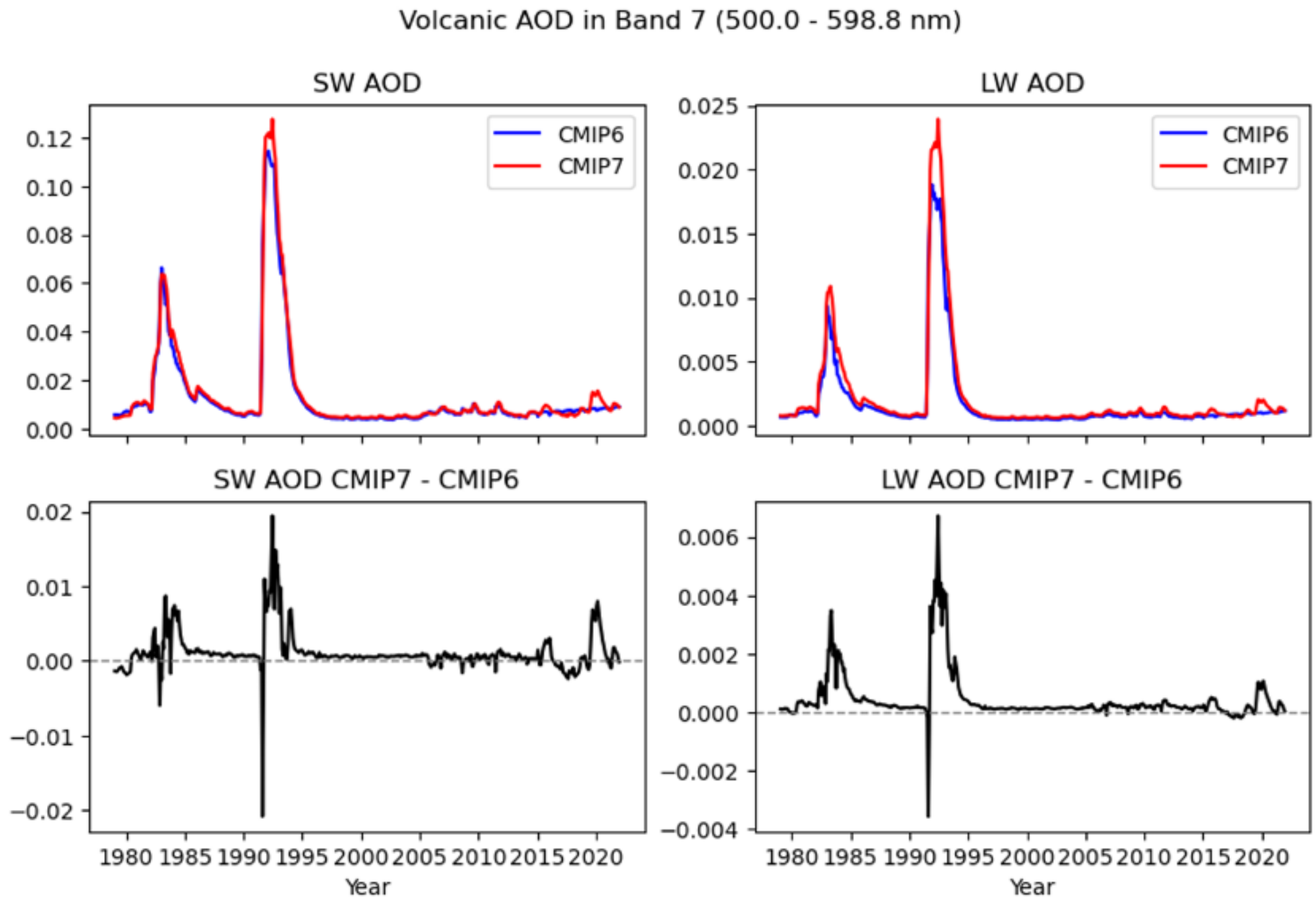

Fig. S3.

Global-mean temporal evolution of stratospheric volcanic shortwave (left) and longwave (right) aerosol optical depth (AOD) in one of the visible bands (500.0~598.8 nm) as inputs to the CMIP6 and CMIP7 simulations. The differences between the two forcing datasets are also plotted in the bottom row.

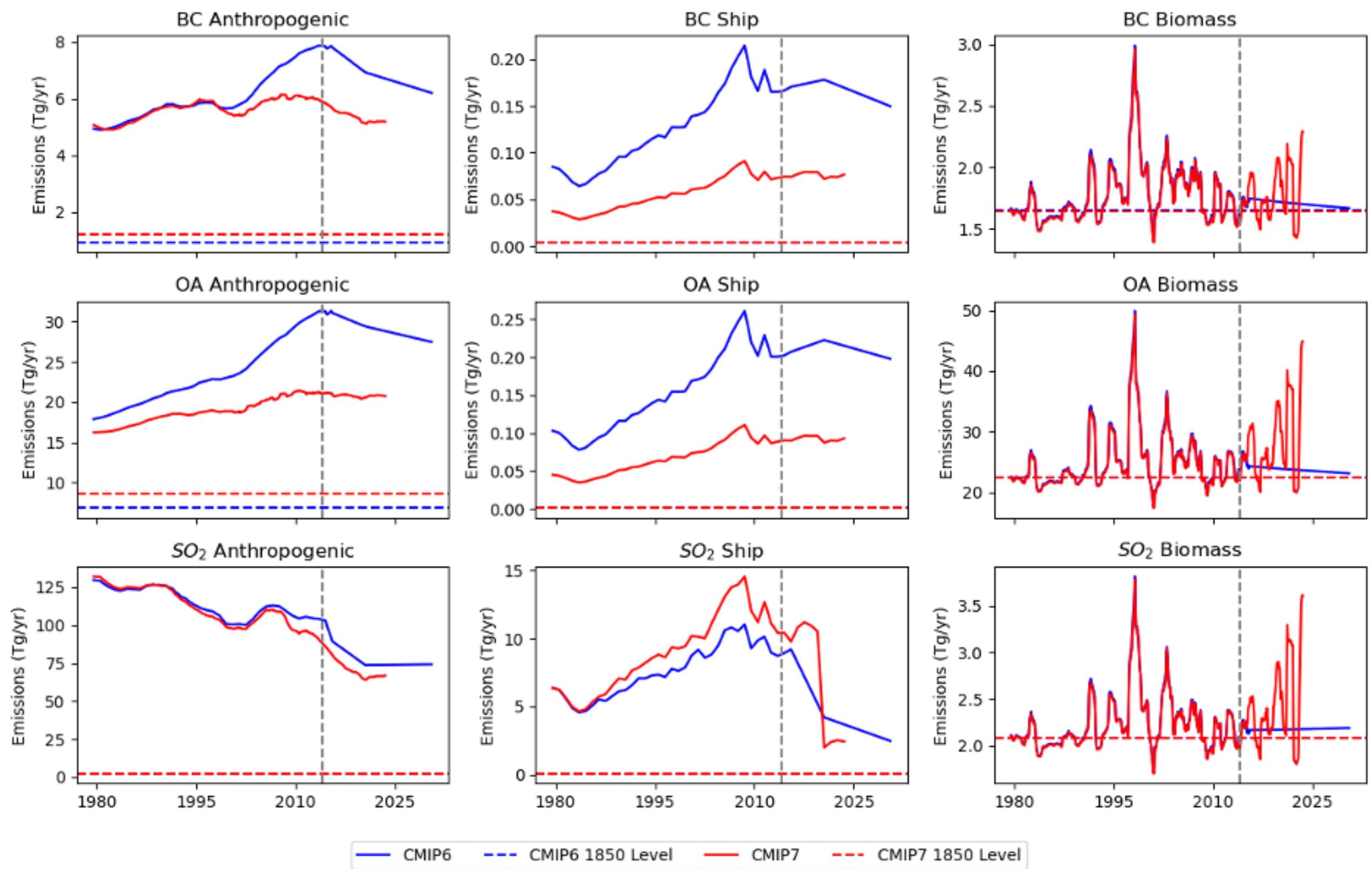

Fig. S4.

Black carbon (BC), organic aerosols (OA), and sulfur dioxide (SO_2) aerosol emissions from biomass burning, anthropogenic sources, and ships based on the CMIP6 and CMIP7 forcing datasets as inputs to the GFDL AM4 simulations. In both the simulation sets, 1% of the SO_2 is emitted as SO_4 to approximate subgrid scale in-plume processing. The gray vertical dashed lines mark the year 2014 after which CMIP6 simulations use SSP2-4.5 scenario forcing.

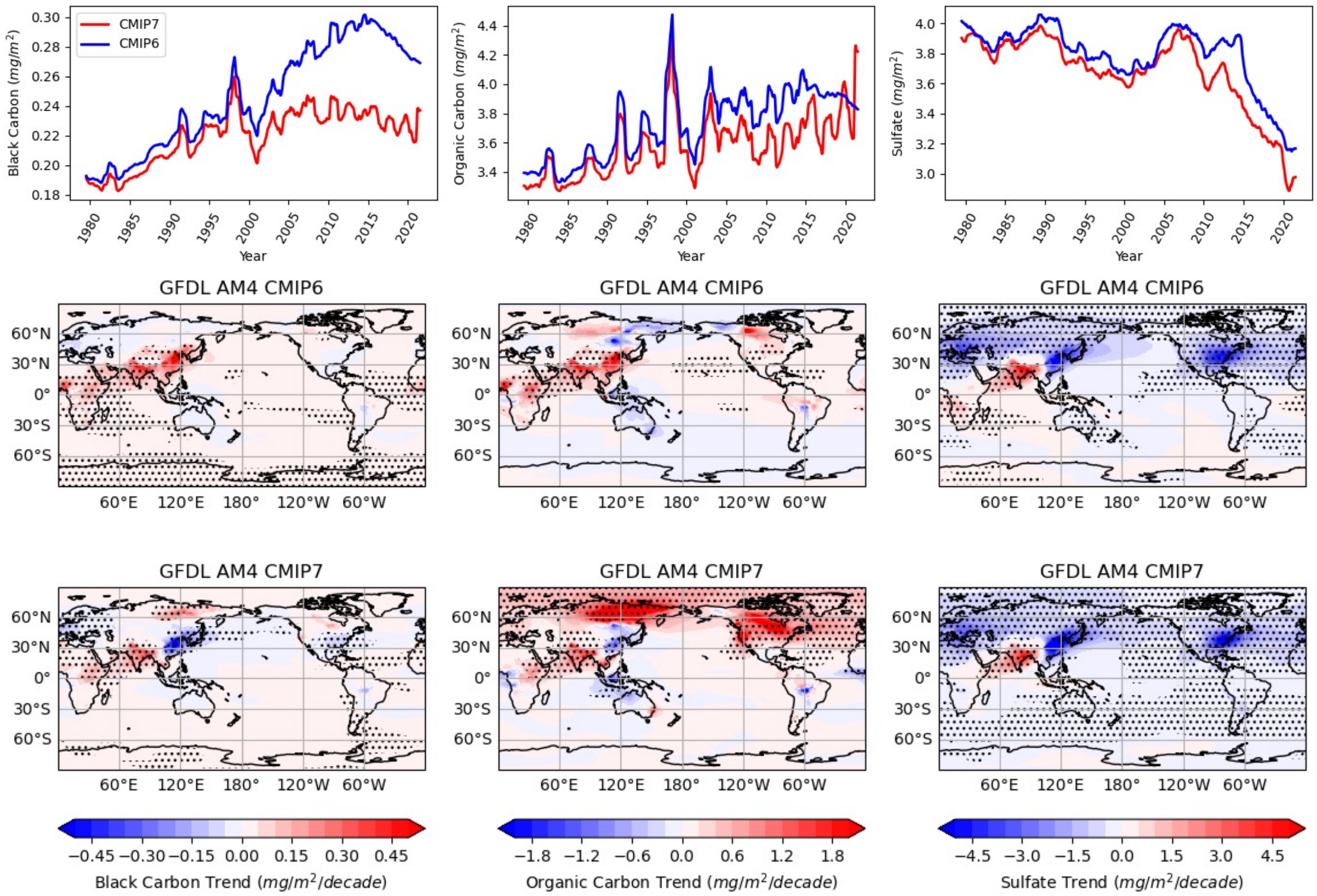

Fig. S5.

(top row) Global mean time series of aerosol loading of black carbon, organic carbon, and sulfate in the CMIP6 (blue) and CMIP7 (red) simulations. (middle row) spatially-resolved linear trends of aerosol loading over the period of 2001~2021 in the CMIP6 simulations. (bottom row) similar to the middle row but showing the trends in the CMIP7 simulations. The dotted region is where the linear trend is significant at 5% significance level.

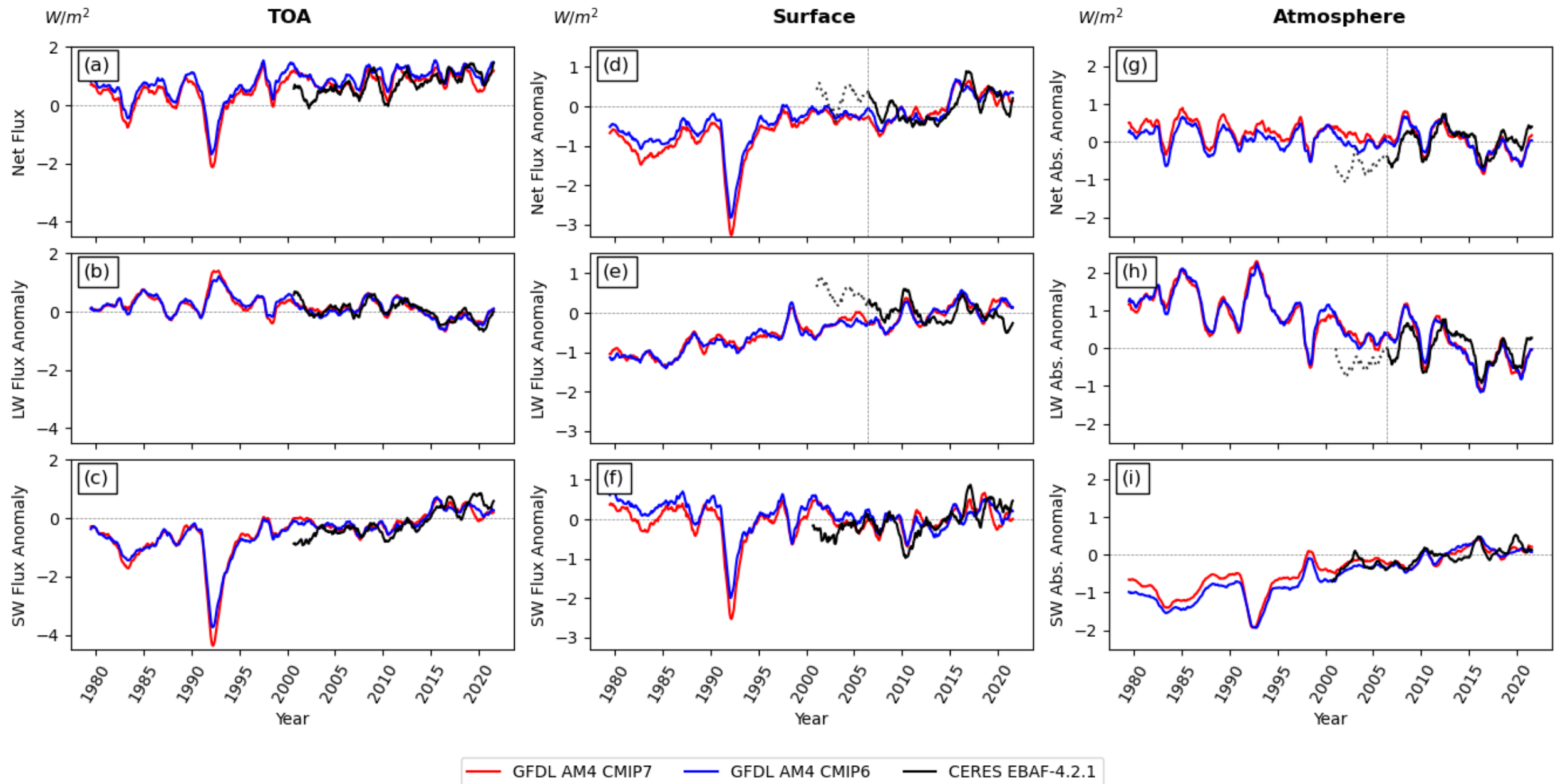

Fig. S6.

Similar to Fig. 1 but showing the full period from 1979 to 2021.

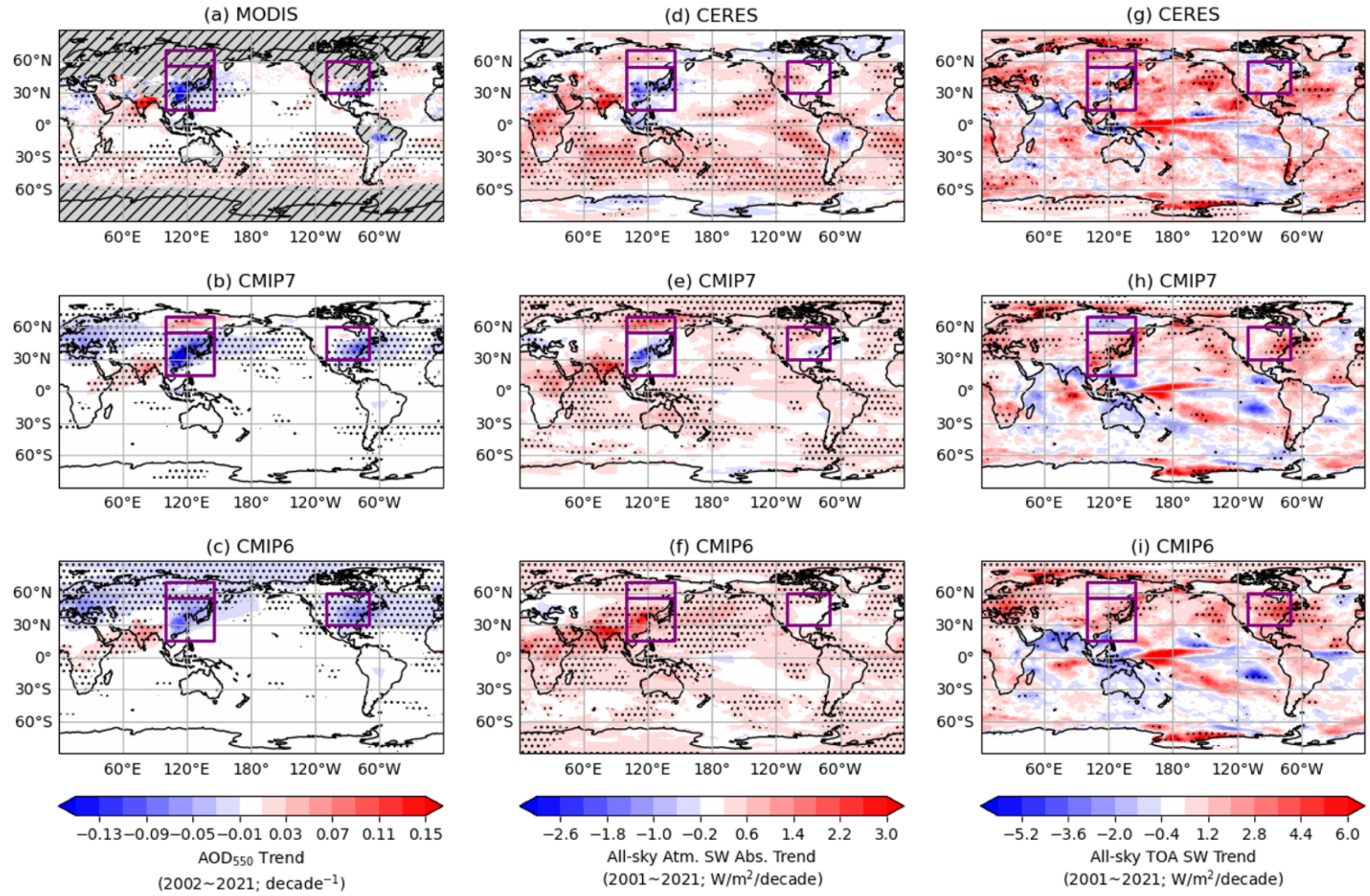

Fig. S7.

Spatial linear trends of aerosol optical depth (AOD) (a-c) over the period of 2002~2021, atmospheric SW absorption (d-f) and TOA SW radiation (g-i) over the period of 2001~2021 from GFDL AM4 using CMIP7 forcing datasets (b,e,h) and CMIP6 forcing datasets (c,f,i) compared to MODIS-observed AOD trends (a) and CERES-derived SW trends (d,g), respectively. Gray hatched masking in MODIS observations (a) indicates that more than 25% of the samples in the time series are missing over these grid points. Black dotted region indicates that the linear trend is statistically significant at 95% confidence level. The purple boxes indicate the three regions of interest discussed in Section 3.

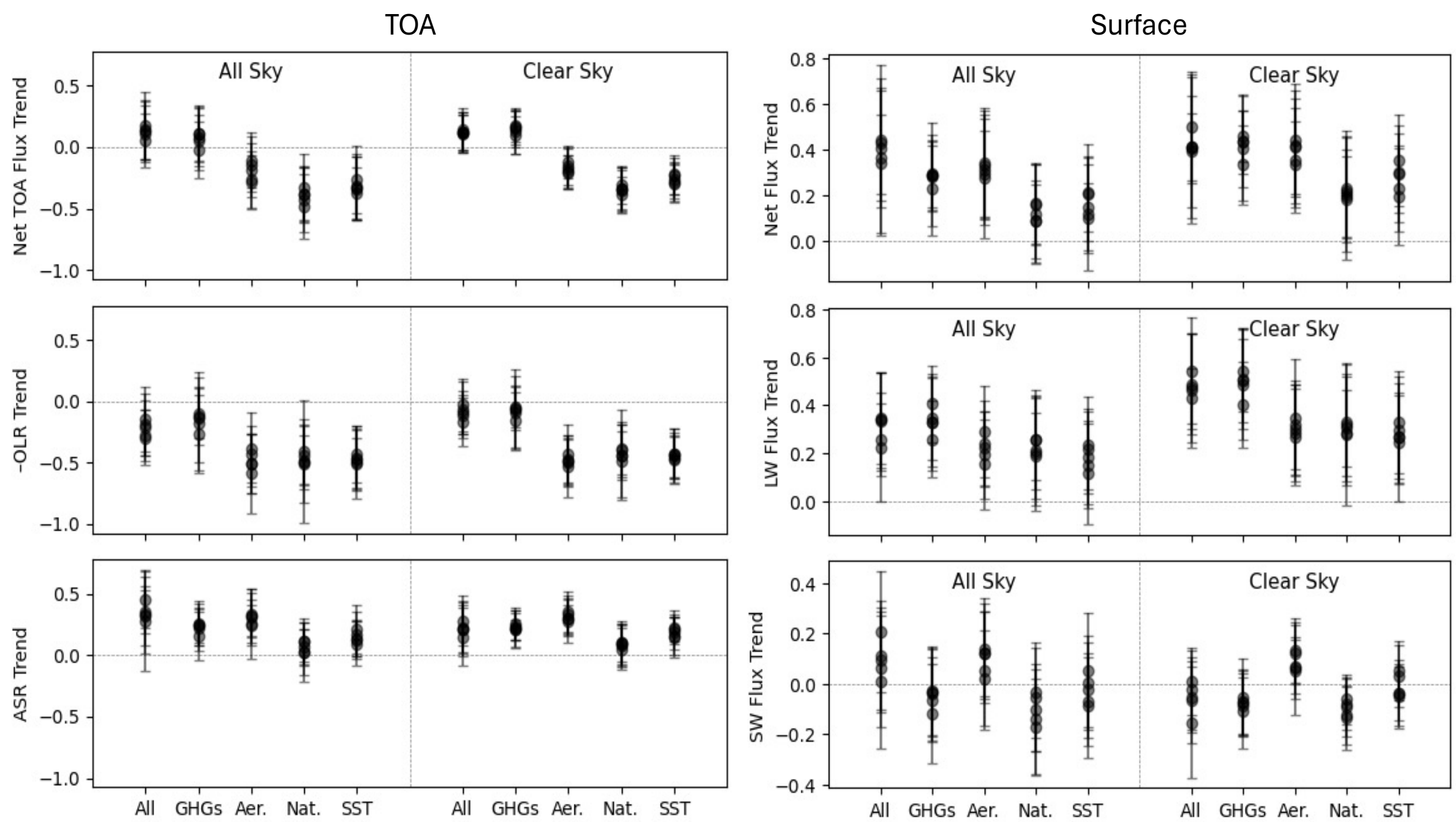

Fig. S8.

Linear trend of TOA and surface radiative fluxes in CMIP7 all-forcing experiment (All) and single-forcing experiments from 2001 to 2021. Left column shows all-sky and clear-sky TOA flux trends, specifically net TOA flux trends (top), –OLR trend (middle), and ASR trend (bottom). Right column shows all-sky and clear-sky surface flux trends, specifically net surface flux trend (top), longwave surface flux trend (middle), and shortwave surface flux trend (bottom). Single-forcing experiments include greenhouse gases only (GHGs), aerosols only (Aer.), natural forcings only (Nat.), and historical SST and sea ice only (SST). Refer to supplementary text for more information. Note that the linear trend of the all-forcing run may not equal to the sum of the linear trends of the single-forcing runs.

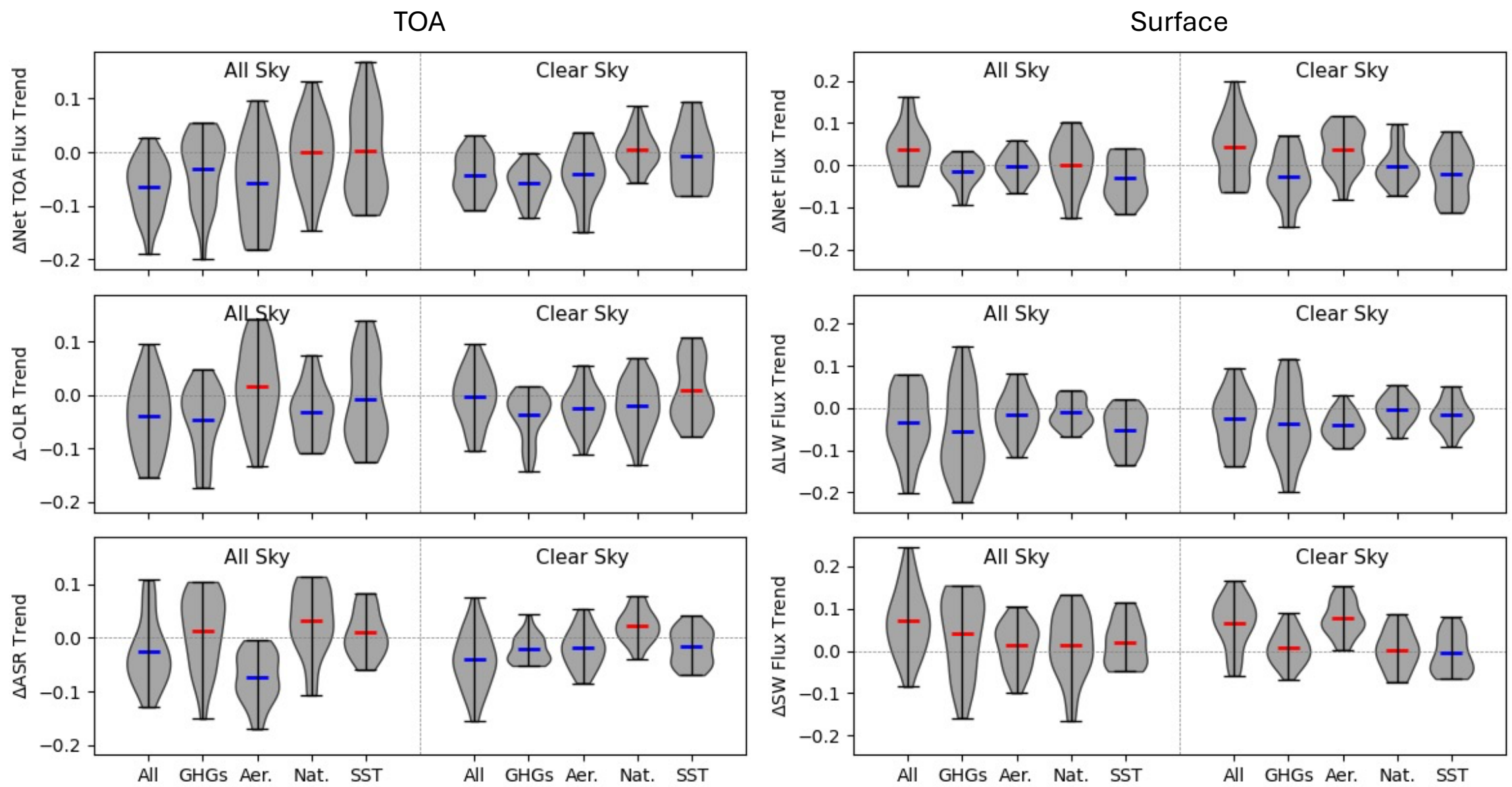

Fig. S9.

Similar to Fig. S8, but showing the differences in linear flux trends between CMIP7 simulations and CMIP6 simulations. The differences are computed by subtracting each of the five ensemble members of CMIP7 simulations by each of the five ensemble members of CMIP6 simulations, totaling 25 pairs. The results are visualized as a violin plot showing the probability distribution.

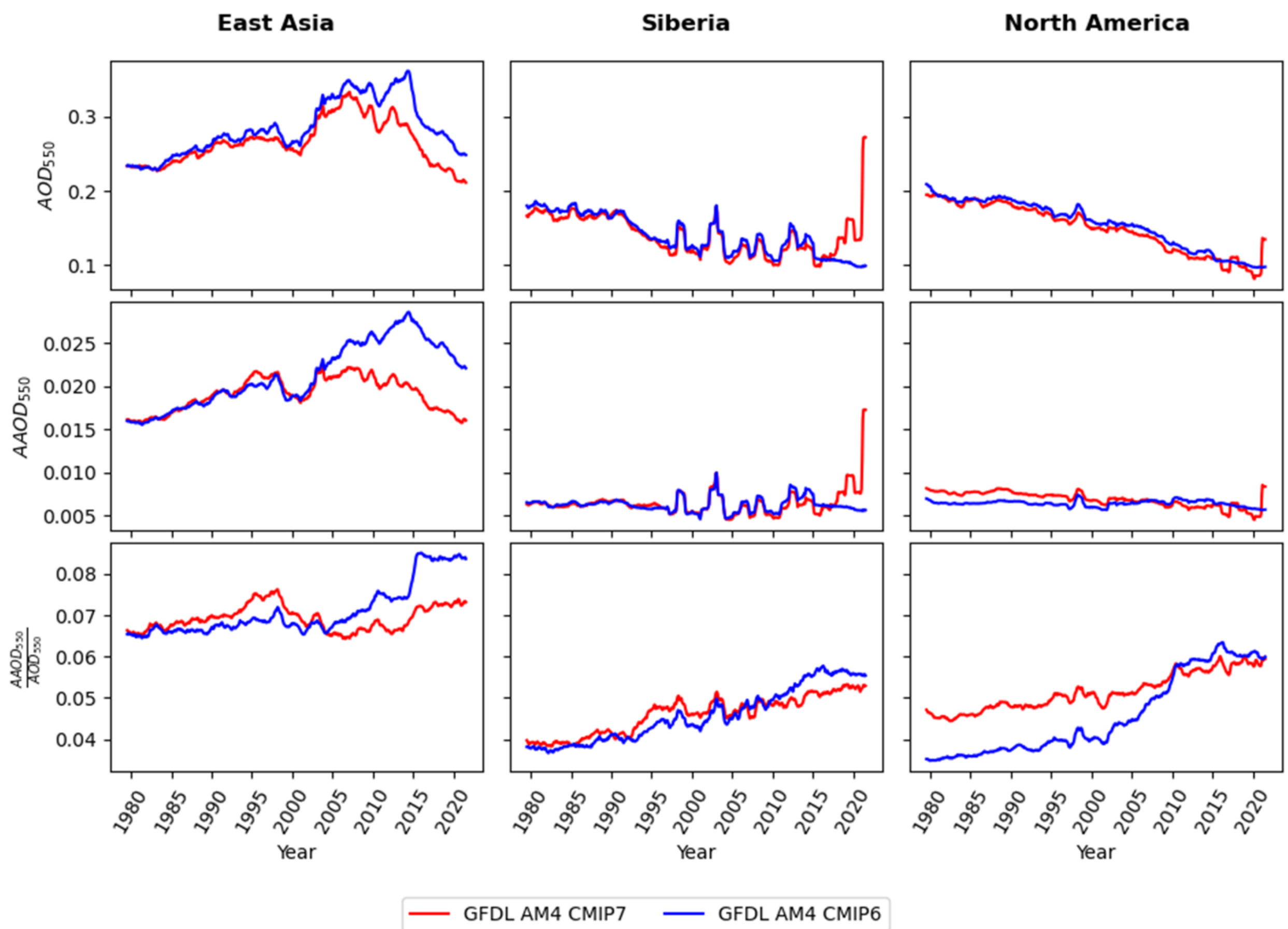

Fig. S10.
Regional mean time series of total aerosol optical depth (AOD) (top row), absorptive AOD (middle row), and the ratio of the two (bottom row) averaged East Asia (left column), East Siberia (middle column), and North America (right column).

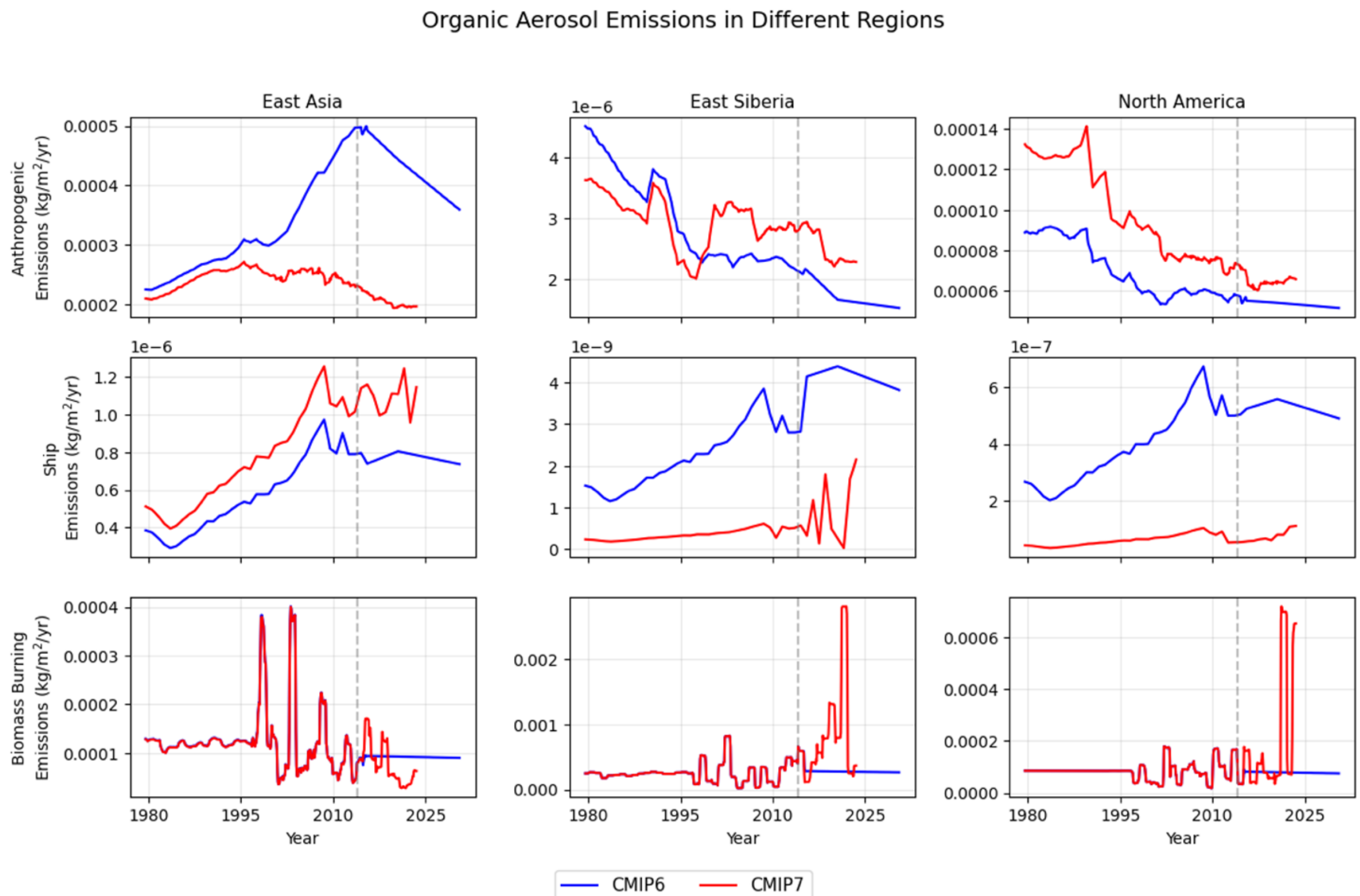

Fig. S11.

Regional-mean time series of organic aerosol emissions from anthropogenic emission (top row), ship emission (middle row), and biomass burning (bottom row) in the CMIP6 and CMIP7 forcing datasets over East Asia, East Siberia, and North America (the same definition as in Figs. 3-4). All the time series are applied 12-month smoothing averages for visualization purposes. The transition between the CMIP6 dataset and the SSP2-4.5 dataset occurs at the end of 2014, as marked by the gray dashed line.

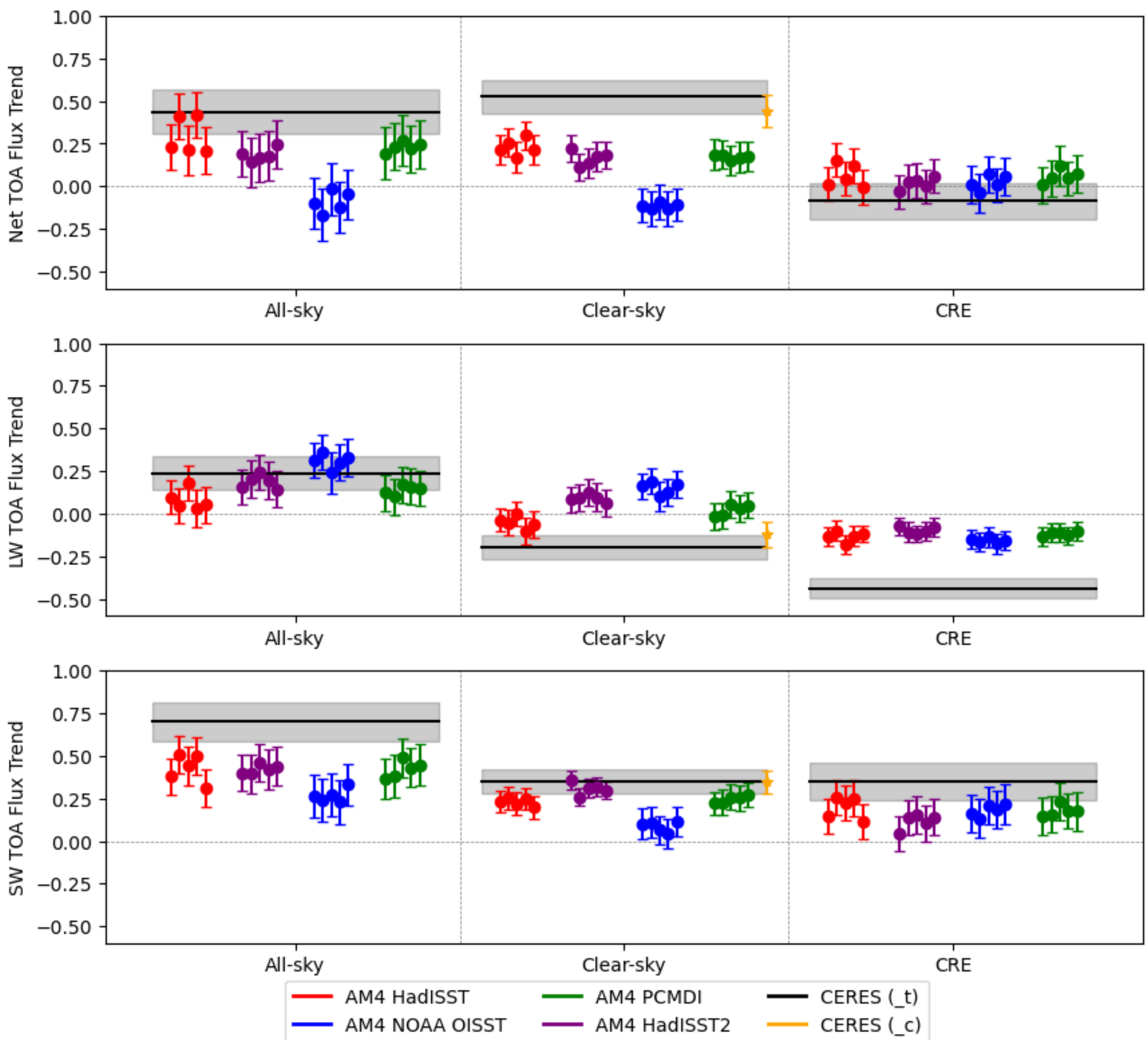

Fig. S12.

Linear trends of simulated TOA radiative fluxes from 2001 to 2021 in GFDL AM4 simulations with different SST and sea ice datasets, reproduced from Fan et al. (1). Each of the three rows shows net TOA flux trend, longwave TOA flux trend (i.e., OLR trend, positive upwards), and shortwave TOA flux trend (i.e., ASR trend). Each column represents all-sky flux trends, clear-sky flux trends, and cloud radiative effect (CRE) trends. Each colored dot of each category (except the single yellow dot) indicates simulated flux trend in each ensemble member with the error bar showing 95% confidence interval. Black lines indicate CERES-observed trends with the shaded region indicating 95% confidence interval. By default, CERES clear-sky flux based on cloud-clearing algorithm is used to compute the clear-sky flux trends. The trend based on real clear sky selection is shown as the single yellow dot in the second column. All simulations in this figure use CMIP6 + SSP2-4.5 forcing datasets.

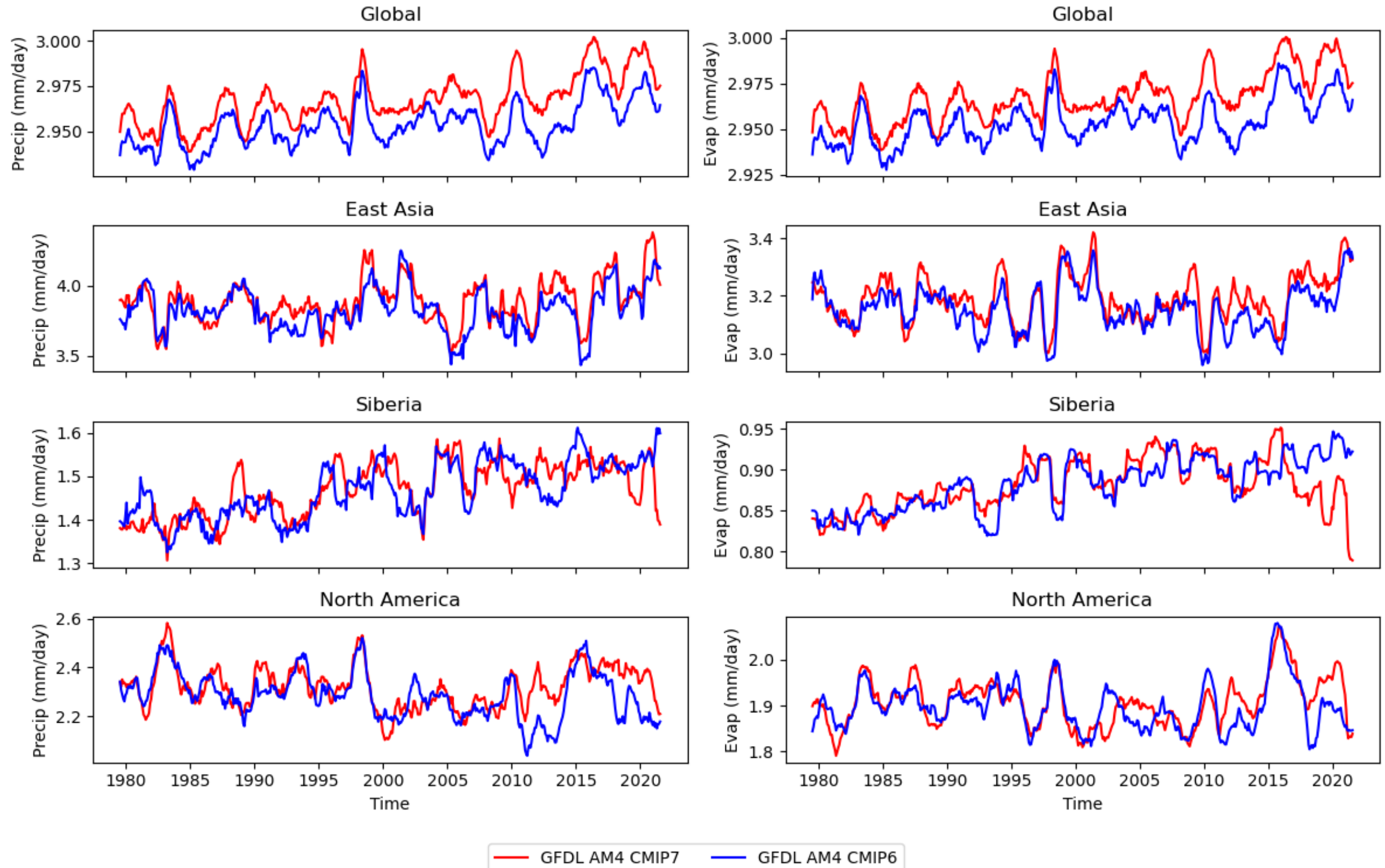

Fig. S13.

Time series of precipitation (left column) and evaporation (right column), averaged over the globe (top row), East Asia (2nd row), East Siberia (3rd row), and North America (bottom row). The regional definitions align with those presented in Figs. 3-4. For visualization, a 12-month moving average is applied to all time series.

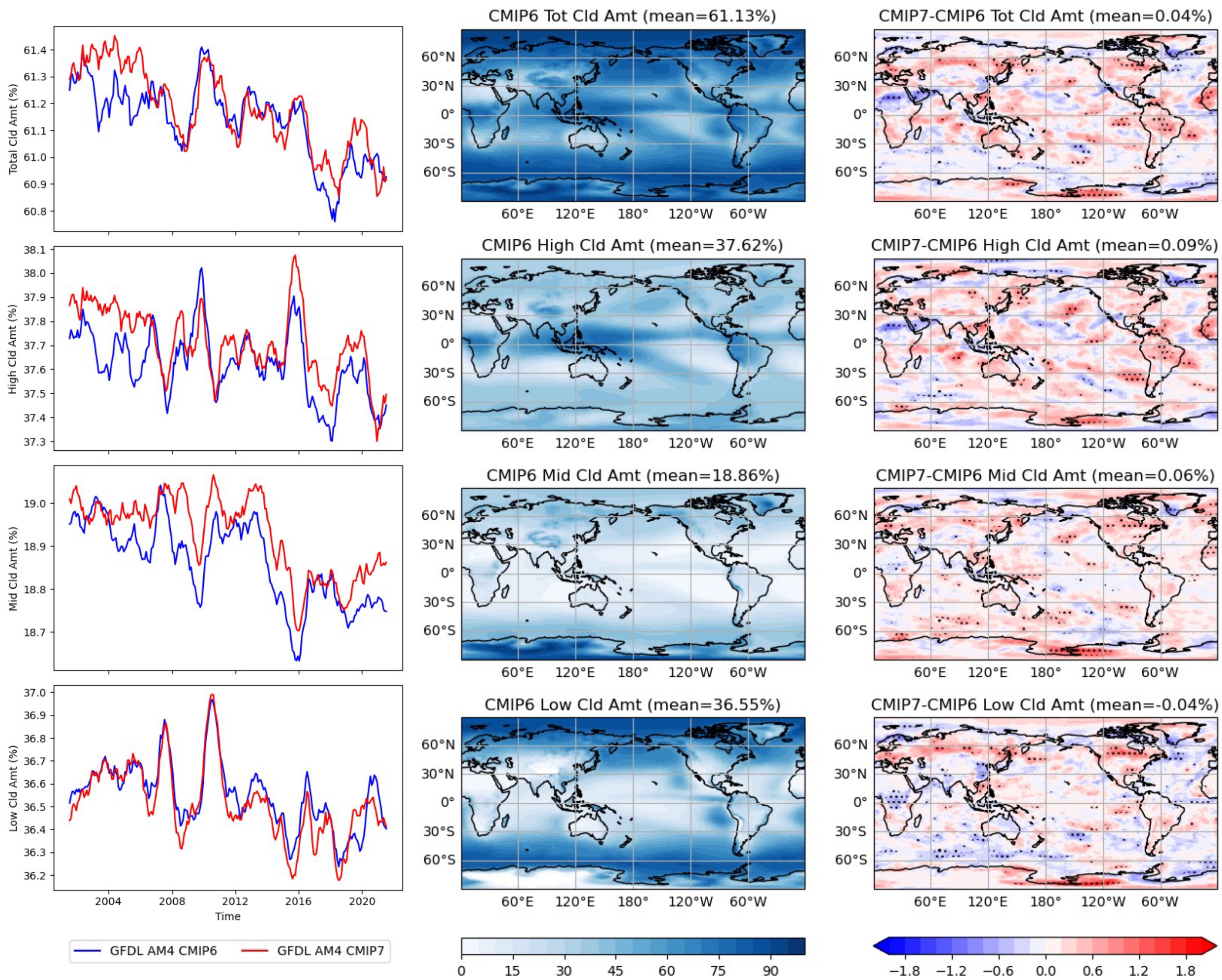

Fig. S14.

Comparison of total, high, middle, and low cloud amounts in GFDL AM4 CMIP6 and CMIP7 simulations. (left column) Global mean time series of total, high, middle, and low cloud cover (%), smoothed using a 12-month moving average. (middle column) Spatially resolved long-term annual means (2001~2021). (right column) Spatial differences between CMIP7 and CMIP6 (CMIP7 − CMIP6). Dotted regions are where differences are statistically significant at 95% confidence level.

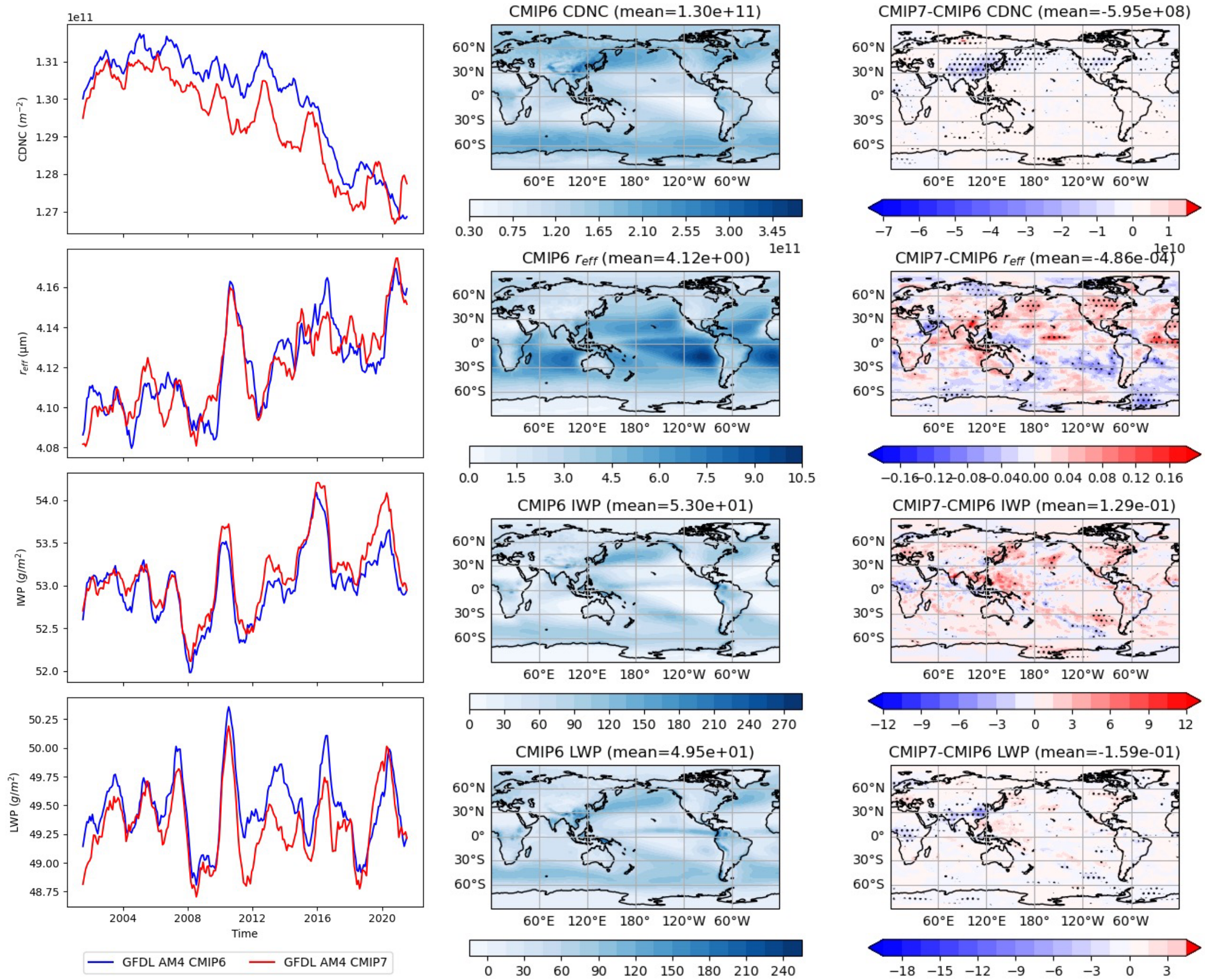

Fig. S15.

Similar to Fig. S14, but evaluating vertically summed cloud droplet number concentration (CDNC; m^{-2}), cloud-top effective droplet radius (r_{eff}; μm), ice water path (IWP; g m^{-2}), and liquid water path (LWP; g m^{-2}).

Tables

Table S1. Linear trends of surface fluxes and atmospheric absorption in CERES observations and the ensemble mean of model simulations over the period of 2006~2021 in the unit of W/m^2/decade. The uncertainty values are 95% confidence intervals. Note that the narrowing SW trend differences between CMIP7 and CMIP6 simulations suggest that the aerosol forcing revision over the 2001-2006 period contributes to the trend differences over the full period (Figure 3a and Figure S4).

	**CERES EBAF Ed4.2.1**	**GFDL AM4 CMIP7**	**GFDL AM4 CMIP6**
		**Surface (2006~2021)**	
**All–sky Net**	0.31 ± 0.54	0.59 ± 0.37	0.59 ± 0.34
**All–sky LW**	–0.22 ± 0.28	0.37 ± 0.27	0.37 ± 0.27
**All-sky SW**	0.53 ± 0.36	0.23 ± 0.30	0.22 ± 0.29
**Clear–sky Net**	0.35 ± 0.51	0.63 ± 0.28	0.66 ± 0.27
**Clear–sky LW**	0.28 ± 0.36	0.58 ± 0.32	0.61 ± 0.30
**Clear-sky SW**	0.08 ± 0.29	0.04 ± 0.22	0.05 ± 0.29
		**Atmospheric Absorption (2006~2021)**	
**All–sky Net**	0.13 ± 0.45	–0.45 ± 0.40	–0.46 ± 0.40
**All–sky LW**	–0.21 ± 0.52	–0.77 ± 0.55	–0.81 ± 0.54
**All-sky SW**	0.35 ± 0.19	0.32 ± 0.20	0.35 ± 0.39
**Clear–sky Net**	0.16 ± 0.46	–0.49 ± 0.40	–0.52 ± 0.38
**Clear–sky LW**	–0.20 ± 0.52	–0.84 ± 0.57	–0.91 ± 0.55
**Clear-sky SW**	0.36 ± 0.21	0.35 ± 0.21	0.39 ± 0.43

Table S2. Linear trends of TOA fluxes averaged over Northern Hemisphere and Southern Hemisphere over the period of 2001~2021 in the unit of W/m^2/decade. The uncertainty values are 95% confidence intervals.

	**CERES EBAF Ed4.2.1**	**GFDL AM4 CMIP7**	**GFDL AM4 CMIP6**
	**TOA – Northern Hemisphere (0~90ºN)**		
**All–sky Net**	0.44 ± 0.25	0.22 ± 0.26	0.40 ± 0.27
**All–sky LW**	–0.34 ± 0.24	–0.40 ± 0.26	–0.28 ± 0.27
**All–sky SW**	0.78 ± 0.26	0.61 ± 0.28	0.68 ± 0.35
**Clear–sky Net**	0.61 ± 0.25	0.13 ± 0.19	0.27 ± 0.20
**Clear–sky LW**	0.07 ± 0.19	–0.24 ± 0.20	–0.20 ± 0.23
**Clear–sky SW**	0.54 ± 0.23	0.37 ± 0.17	0.46 ± 0.18
	**TOA – Southern Hemisphere (0~90ºS)**		
**All–sky Net**	0.43 ± 0.29	0.03 ± 0.25	–0.03 ± 0.21
**All–sky LW**	–0.13 ± 0.24	–0.06 ± 0.29	–0.10 ± 0.41
**All–sky SW**	0.57 ± 0.26	0.08 ± 0.26	0.06 ± 0.25
**Clear–sky Net**	0.45 ± 0.28	0.11 ± 0.22	0.06 ± 0.20
**Clear–sky LW**	0.33 ± 0.17	0.05 ± 0.21	0.01 ± 0.22
**Clear–sky SW**	0.11 ± 0.25	0.06 ± 0.23	0.05 ± 0.21

SI References

1. C. Fan, D. Paynter, R. J. Kramer, P. Lin, Sensitivity of Earth's Radiation Budget to Lower Boundary Condition Data Sets in Historical Climate Simulations. *Geophysical Research Letters* **52**, e2025GL115914 (2025).

2. G. A. Schmidt, *et al.*, CERESMIP: a climate modeling protocol to investigate recent trends in the Earth's Energy Imbalance. *Front. Clim.* **5**, 1202161 (2023).
